# The NOTCH3 extracellular domain is a serum biomarker for pulmonary arterial hypertension

**DOI:** 10.1038/s41591-025-04134-3

**Published:** 2026-01-09

**Authors:** Moises Hernandez, Nolan M. Winicki, Cristian D. Puerta, Swetha Lakshminarayanan, Yu Zhang, Israel Ramirez-Sanchez, Casandra E. Besse, Lin Liu, David S. Poch, Jason X.-J. Yuan, John Y.-J. Shyy, Paul B. Yu, Joe G. N. Garcia, Patricia A. Thistlethwaite

**Affiliations:** 1 Division of Cardiothoracic Surgery, University of California San Diego, La Jolla, CA USA; 2 National Polytechnic Institute, Higher Education School of Medicine Graduate Studies and Research, Mexico City, Mexico; 3 Division of Biostatistics and Bioinformatics, Herbert Wertheim School of Public Health and Human Longevity Science, University of California San Diego, La Jolla, CA USA; 4 Division of Pulmonary Medicine, University of California San Diego, La Jolla, CA USA; 5 University of Florida Scripps Institute for Biomedical Innovation and Technology, Jupiter, FL USA; 6 Institute of Engineering in Medicine, University of California San Diego, La Jolla, CA USA; 7 Division of Cardiology, Massachusetts General Hospital, Boston, MA USA

**Keywords:** Diagnostic markers, Prognostic markers

## Abstract

New biomarkers are needed to detect and follow individuals with World Health Organization group 1.1 pulmonary hypertension (idiopathic pulmonary arterial hypertension (IPAH)). As NOTCH3 cleavage occurs constitutively in the lungs of individuals with IPAH, we investigated whether the NOTCH3 extracellular domain (NOTCH3-ECD) shed into serum could be used as a robust biomarker for IPAH. In three geographically distinct cohorts comprising 341 individuals with IPAH (267 women, 74 men) and 376 healthy individuals (278 women, 98 men), serum NOTCH3-ECD levels were significantly higher in individuals with IPAH (mean ± s.d.: 19.9 ± 5.5 ng ml^−1^) compared to controls (10.5 ± 1.9 ng ml^−1^; *P* < 0.001), with consistent results among the three cohorts. NOTCH3-ECD levels correlated with mean right atrial pressure, pulmonary vascular resistance, mean pulmonary artery pressure, tricuspid regurgitant velocity, 6-min walk distance and the New York Heart Association class. The area under the receiver operating curve for diagnosis of IPAH, based on serum NOTCH3-ECD, was 0.96 (95% confidence interval, 0.95–0.98) with a 90% sensitivity and 93% specificity at a cutoff of 13.0 ng ml^−1^. The 3-year mortality risk for individuals with IPAH increased by 18% for each increase in 3 ng ml^−1^ of NOTCH3-ECD above the diagnostic cutoff. The addition of serum NOTCH3-ECD levels improved the performance of prognostic calculators for PAH, including REVEAL 2.0, REVEAL 2.0 Lite and COMPERA 2.0. Moreover, serum NOTCH3-ECD levels predicted the presence of IPAH in treatment-naive individuals and correlated with disease progression over a follow-up of 6 years. Measurement of serum NOTCH3-ECD can therefore provide a highly sensitive, specific and noninvasive test for predicting the presence, disease severity, progression and survival of individuals with IPAH.

## Abstract (web summary)

A protein biomarker, the NOTCH3 extracellular domain, identifies individuals with idiopathic pulmonary hypertension, correlates with disease progression, improves mortality risk prediction and provides a readily implementable, noninvasive blood test for this disease.

## Main

Idiopathic pulmonary arterial hypertension (IPAH: World Health Organization (WHO) group 1.1) is defined by sustained elevation of mean pulmonary arterial pressure (mPAP > 20 mm Hg) and pulmonary vascular resistance (PVR > 2 Wood units) in the setting of pulmonary artery wedge pressure (PAWP ≤ 15 mm Hg), leading to right ventricular failure and death^1,2^. At the cellular level, this pathological process is characterized by increased vascular smooth muscle cell (vSMC) burden^3^, neointimal hyperplasia^4^ and perivascular inflammation in small pulmonary arteries^5^. Currently, IPAH is diagnosed by a careful history and physical examination, chest radiography, electrocardiogram, echocardiography (ECHO), right heart catheterization (RHC), arterial blood gas and serum N-terminal pro-B-type natriuretic peptide or B-type natriuretic peptide (NT-proBNP or BNP) testing, as well as ventilation–perfusion (V/Q) scanning, chest computed tomography and pulmonary function testing to rule out other diseases^1,2^. Once treatment begins, individuals are followed with noninvasive measures including 6-min walk distance (6MWD), New York Heart Association (NYHA) functional class, NT-proBNP or BNP levels, pulse oximetry, health-related quality of life questionnaires and ECHO^1,2,6^. RHC with measurement of defined parameters^1,2^ is performed at diagnosis and when treatment escalation may be considered. These tests are expensive (RHC, ECHO, V/Q scan, computed tomography), invasive and potentially painful (RHC) and lack specificity (NT-proBNP, BNP, NYHA class, 6MWD). A major barrier to the diagnosis and treatment of IPAH is the lack of a noninvasive blood test that is highly sensitive and specific to diagnose and follow the course of individuals with this disease.

Several lines of evidence suggest that NOTCH3 signaling is central to the development of IPAH. NOTCH3 is uniquely expressed on vSMCs from small resistance pulmonary arteries in human and rodent lungs^7^. Within the pulmonary vasculature, NOTCH3 signaling is initiated by the binding of the selective NOTCH ligand, JAGGED-1 (JAG-1), to the receptor, stimulating cleavage of the receptor into two peptides: an intracellular domain (NOTCH3-ICD) and an extracellular domain (NOTCH3-ECD)^8^. The ICD translocates to the nucleus and functions as a transcriptional enhancer of *hairy/enhancer* *of split* (*Hes*) and *hairy/enhancer of split-related* (*Hrt*) genes, the protein products of which modulate the transcription of genes involved in cellular homeostasis and proliferation^9^. Small pulmonary artery smooth muscle cells (sPASMCs: <500 μm in diameter) from individuals with IPAH have strong autocrine production of JAG-1, which stimulates NOTCH3 receptor cleavage in these cells^10^. Human IPAH lung tissue is characterized by high levels of NOTCH3-ICD in sPASMCs, and disease severity in humans correlates with the amount of NOTCH3-ICD protein in the lung^7^. Mice with homozygous deletion of *Notch3* do not develop pulmonary hypertension (PH) in response to hypoxic stimulation^7^ and PH can be successfully treated in rats by administration of a monoclonal antibody that specifically inhibits JAG-1 ligand binding to the NOTCH3 receptor, inhibiting downstream signaling^10^. Recently, the cells that cause neointimal thickening, which contribute to occlusive pulmonary vascular lesions in rodent PH, have been identified by lineage tracing as NOTCH3-expressing subpopulations of sPASMCs^11^. Despite extensive study of NOTCH3-ICD in the lungs of individuals with IPAH, little is known about the fate of NOTCH3-ECD after receptor activation and cleavage.

In this study, we investigated whether NOTCH3-ECD is shed into the serum of patients with IPAH. We report the performance of serum NOTCH3-ECD as a robust biomarker for diagnosis, indicator of disease severity and progression, and risk of mortality for individuals with this morbid disease.

## Results

### Serum NOTCH3-ECD levels correlate with IPAH disease severity

**Fig. 1:**
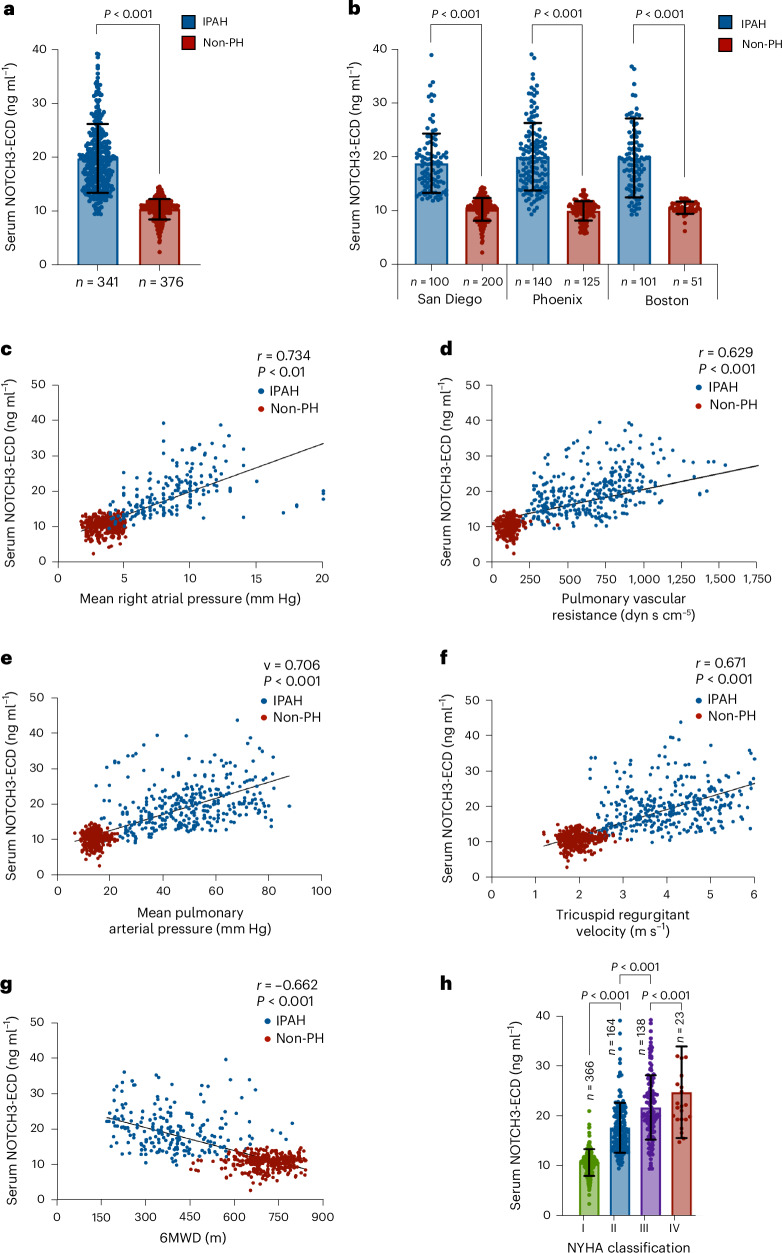
NOTCH3-ECD serum levels predict the presence of IPAH and correlate with IPAH disease parameters. **a**, Mean serum levels of NOTCH3-ECD in patients with IPAH (*n* = 341) and individuals without PH (*n* = 376) from the combined San Diego, Phoenix and Boston cohorts (*P* < 0.001*)*. **b**, Mean serum levels of NOTCH3-ECD in patients with IPAH and individuals without PH from the individual cohorts (*P* < 0.001 for each cohort). San Diego cohort: *n* = 100 (IPAH), *n* = 200 (non-PH); Phoenix cohort: *n* = 140 (IPAH), *n* = 125 (non-PH); and Boston cohort: *n* = 101 (IPAH), *n* = 51 (non-PH). **c**–**g**, Correlations between serum NOTCH3-ECD levels and mRAP (*P* < 0.01) (**c**), PVR (*P* < 0.001) (**d**), mPAP (*P* < 0.001) (**e**), TRV (*P* < 0.001) (**f**) and 6MWD (*P* < 0.001) (**g**) in the combined cohort. **h**, Mean serum levels of NOTCH3-ECD relative to NYHA class in individuals with IPAH compared to individuals without PH (*P* < 0.001). Each dot in **a**–**h** represents a single individual. The two-sided *P* values in **a** and **b** were determined using an independent two-sample Student’s *t*-test. For dot plots in **c**–**g**, *r* and *P* values were calculated using Spearman’s rank correlation. The two-sided *P* values in **h** were determined by ANOVA with post-hoc Tukey tests. The data in **a**, **b** and **h** are expressed as mean ± s.d. Source data

**Extended Data Fig. 1:**
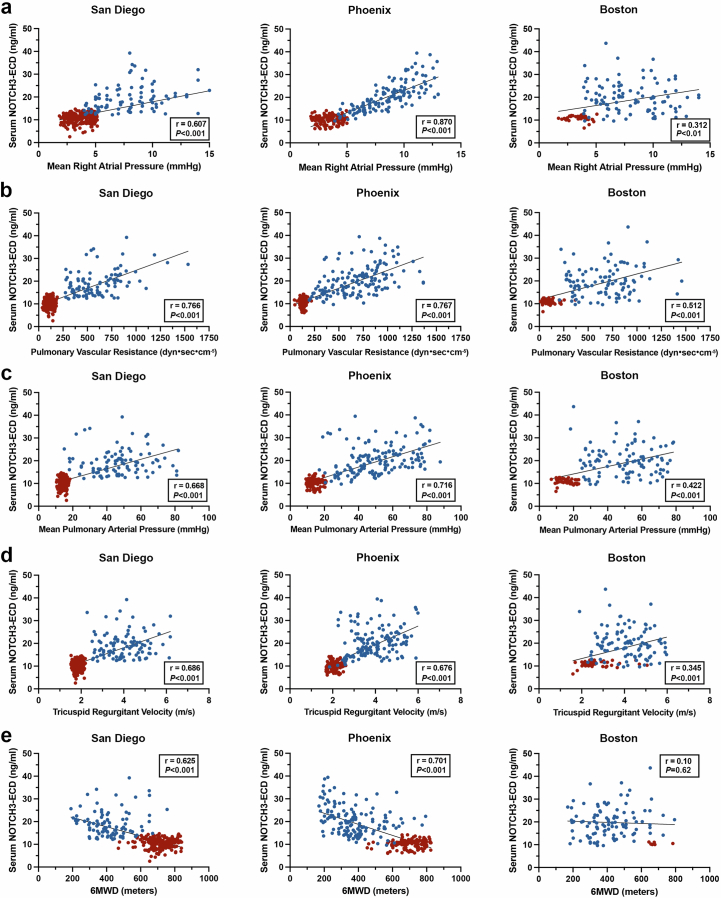
NOTCH3-ECD serum levels correlate with idiopathic pulmonary arterial hypertension (IPAH) disease parameters in three geographically different cohorts. **a-e**, The correlation between serum NOTCH3-ECD levels and mean right atrial pressure (mRAP; *P* < 0.001 for San Diego and Phoenix cohorts, *P* < 0.01 for Boston cohort) (**a**), pulmonary vascular resistance (PVR; all *P* < 0.001) (**b**), mean pulmonary artery pressure (mPAP; all *P* < 0.001) (**c**), tricuspid regurgitant velocity (TRV; all *P* < 0.001) (**d**), and 6-minute walk distance (6MWD; *P* < 0.001 for San Diego and Phoenix cohorts, *P* = 0.62 for Boston cohort) (**e**), San Diego cohort: n = 100 (IPAH), n = 200 (non-PH); Phoenix cohort: n = 140 (IPAH), n = 125 (non-PH); Boston cohort: n = 101 (IPAH), n = 51 (non-PH). Each blue dot represents a single individual with IPAH, while each red dot indicates an individual without PH in **a-e**. For dot plots in **a-e**, r and *P* values were calculated using Spearman rank correlation.

**Extended Data Fig. 2:**
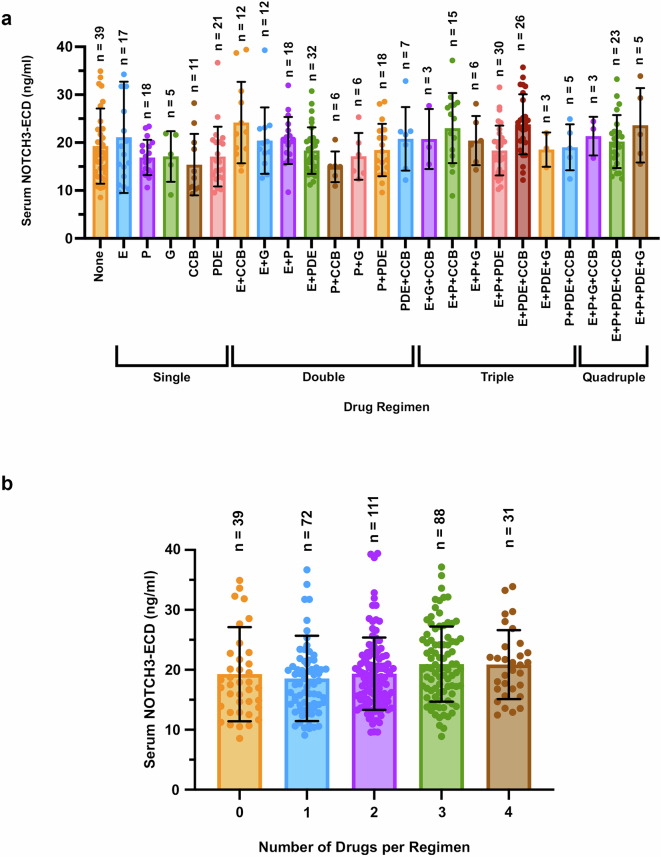
Serum NOTCH3-ECD levels did not differ between individuals receiving varying drug regimens or number of PAH drugs. **a**, Serum NOTCH3-ECD levels in individuals treated with different PAH-specific drug regimens. **b**, Serum NOTCH3-ECD levels in individuals treated with different number of drugs per regimen (no drug regimen: n = 39, single-drug regimen: n = 72, two-drug regimen: n = 111, three-drug regimen: n = 88, four-drug regimen: n = 31). Each dot represents a single individual. The data in **a** and **b** are expressed as mean ± s.d. E = endothelin receptor antagonist, P = prostacyclin analog, G = guanylate cyclase stimulator, CCB = calcium channel blocker, PDE = phosphodiesterase-5-inhibitor.

Three hundred forty-one non-genotyped patients with previously diagnosed IPAH and 376 non-PH individuals had a serum sample collected (combined cohort: San Diego, Phoenix and Boston) with a median follow-up time of 4.75 years (interquartile range (IQR), 2.89–5.35 years) (Table 1). Mean serum NOTCH3-ECD levels were significantly higher for patients with IPAH (mean ± s.d., 19.9 ± 5.5 ng ml^−1^) compared to individuals without PH (10.5 ± 1.9 ng ml^−1^; *P* < 0.001) (Fig. 1a). In the individual San Diego, Phoenix and Boston cohorts (Table 1), the mean serum NOTCH3-ECD levels were similar between individuals with IPAH (mean ± s.d., 19.2 ± 5.0 ng ml^−1^, 20.3 ± 5.9 ng ml^−1^ and 20.2 ± 7.0 ng ml^−1^, respectively; *P* = 0.33) (Fig. 1b). The mean serum NOTCH3-ECD levels were also similar between individuals without PH in the San Diego, Phoenix and Boston cohorts (mean ± s.d., 10.6 ± 1.4 ng ml^−1^, 10.2 ± 2.2 ng ml^−1^ and 10.9 ± 1.4 ng ml^−1^, respectively; *P* = 0.70), with a statistically significant difference in serum NOTCH3-ECD levels between individuals with IPAH and individuals without PH in each cohort (*P* < 0.001) (Fig. 1b). For the combined three-cohort group, NOTCH3-ECD serum levels correlated with the degree of: mRAP (*r* = 0.734, *P* < 0.01); PVR (*r* = 0.629, *P* < 0.001); mPAP (*r* = 0.706, *P* < 0.001); TRV (*r* = 0.671, *P* < 0.001); 6MWD (*r* = −0.662, *P* < 0.001); and NT-proBNP (*r* = 0.258, *P* < 0.001) (Fig. 1c–g). In addition, increasing levels of NYHA class were associated with higher serum NOTCH3-ECD: NYHA class I: 10.8 ± 2.7 ng ml^−1^; NYHA class II: 17.8 ± 4.9 ng ml^−1^; NYHA class III: 21.8 ± 6.4 ng ml^−1^; and NYHA class IV: 24.9 ± 8.9 ng ml^−1^ (*P* < 0.001) (Fig. 1h). This trend was further supported by significant correlations between serum NOTCH3-ECD and disease severity parameters for each individual cross-sectional cohort (Extended Data Fig. 1). Serum NOTCH3-ECD levels did not differ between specific patient drug regimens (Extended Data Fig. 2a) or the number of PH drugs used per patient (Extended Data Fig. 2b).

**Table 1:** IPAH and control cross-sectional cohorts

Variable	Combined cohort			San Diego cohort			Phoenix cohort			Boston cohort		
Demographic and clinical characteristics	IPAH (*n* = 341)	Controls (*n* = 376)	*P* value	IPAH^a^ (*n* = 100)	Controls^b^ (*n* = 200)	*P* value	IPAH^a^ (*n* = 140)	Controls^b^ (*n* = 125)	*P* value	IPAH^c^ (*n* = 101)	Controls^d^ (*n* = 51)	*P* value
Age, years	52.7 ± 14.8	51.9 ± 9.7	0.41	54.3 ± 13.0	52.1 ± . 8.5	0.08	53.6 ± 15.4	52.3 ± 11.2	0.44	50.0 ± 14.1	50.5 ± 13.6	0.83
Female sex, *n* (%)	267 (78)	278 (74)	0.19	82 (82)	148 (74)	0.15	105 (75)	93 (74)	0.23	80 (79)	35 (68)	0.16
**Race,** ***n*** **(%)**												
American Indian	6 (1.8)	27 (7.2)		0 (0)	3 (1.5)		6 (4.3)	24 (19.2)		0 (0)	0 (0)	
Asian	10 (2.9)	28 (7.4)		7 (7.0)	16 (8.0)		2 (1.4)	5 (4.0)		1 (1.0)	7 (13.7)	
Black or African American	9 (2.6)	57 (15.2)		2 (2.0)	9 (4.5)		3 (2.1)	31 (24.8)		4 (4.0)	17 (33.3)	
Native Hawaiian or Pacific Islander	1 (0.3)	2 (0.5)		0 (0)	2 (1.0)		1 (0.7)	0 (0)		0 (0)	0 (0)	
More than one race	37 (10.9)	73 (19.4)		29 (29.0)	65 (32.5)		2 (1.4)	7 (5.6)		6 (5.9)	1 (2.0)	
White	264 (77.4)	163 (43.4)		62 (62.0)	96 (48.0)		112 (80.0)	43 (34.4)		90 (89.1)	24 (47.1)	
Unknown or not reported	14 (4.1)	26 (6.9)		0 (0)	9 (4.5)		14 (10.0)	15 (12.0)		0 (0)	2 (3.9)	
BMI, kg m^−2^	29.4 ± 7.4	30.0 ± 3.9	0.18	28.9 ± 8.0	29.4 ± 4.2	0.48	30.0 ± 8.3	29.7 ± 4.5	0.76	29.0 ± 8.0	34.7 ± 8.6	<0.001
Time from RHC to blood collection (days)	16.0 ± 12.1	0 ± 0^e^		24 ± 5.9	0 ± 0^e^		24.1 ± 4.5	0 ± 0^e^		0 ± 0.0^e^	0 ± 0^e^	
**PAH medication regimens**			<0.001			<0.001			<0.001			<0.001
Single, *n* (%)	73 (21)	6 (1.6)		16 (16)	0 (0)		30 (21)	0 (0)		27 (27)	6 (12)	
Double, *n* (%)	111 (33)	0 (0)		48 (48)	0 (0)		29 (21)	0 (0)		34 (34)	0 (0)	
Triple, *n* (%)	87 (26)	0 (0)		31 (31)	0 (0)		42 (30)	0 (0)		14 (14)	0 (0)	
Quadruple, *n* (%)	31 (9)	0 (0)		2 (2)	0 (0)		22 (16)	0 (0)		7 (7)	0 (0)	
None, *n* (%)	39 (11)	370 (98.4)		3 (3)	200 (100)		17 (12)	125 (100)		19 (19)	45 (88)	
**NYHA class**			<0.001			<0.001			<0.001			<0.001
I, *n* (%)	16 (5)	350 (96)		8 (8)	190 (95)		2 (1)	122 (98)		6 (6)	38 (75)	
II, *n* (%)	164 (48)	24 (6)		48 (48)	10 (5)		63 (45)	3 (2)		53 (52)	11 (22)	
III, *n* (%)	138 (40)	2 (1)		40 (40)	0 (0)		60 (43)	0 (0)		38 (38)	2 (4)	
IV, *n* (%)	23 (7)	0 (0)		4 (4)	0 (0)		15 (11)	0 (0)		4 (4)	0 (0)	
6MWD, m	395.9 ± 131.1	708.3 ± 71.8	<0.001	411.4 ± 117.0	709.9 ± 69.3	<0.001	374.6 ± 132.5	706.9 ± 77.1	<0.001	409.9 ± 136.7	683.7 ± 46.4	<0.001
**PAH risk score calculators**												
REVEAL 2.0	6.6 ± 3.0	NR		6.7 ± 3.4	NR		6.7 ± 2.9	NR		6.3 ± 2.8	NR	
REVEAL Lite 2	5.6 ± 2.5	NR		5.7 ± 2.9	NR		5.6 ± 2.4	NR		5.6 ± 2.2	NR	
COMPERA 2.0	1.9 ± 0.8	NR		1.9 ± 0.9	NR		1.8 ± 0.7	NR		1.9 ± 0.9	NR	
mRAP, mm Hg	8.4 ± 3.7	3.6 ± 0.0	<0.001	8.7 ± 4.0	3.7 ± 1.4	<0.001	8.6 ± 2.4	3.5 ± 1.1	<0.001	7.9 ± 3.0	NR	-
mPAP, mm Hg	49.3 ± 16.6	14.6 ± 1.9	<0.001	46.5 ± 15.0	14.5 ± 1.4	<0.001	50.7 ± 16.6	14.4 ± 3.4	<0.001	50.0 ± 16.1	15.4 ± 4.3	<0.001
PAWP, mm Hg	10.8 ± 3.7	8.7 ± 1.9	<0.001	11.2 ± 5.0	8.1 ± 2.8	<0.001	10.3 ± 3.6	9.4 ± 1.1	<0.001	11.0 ± 4.0	10.0 ± 1.4	<0.001
PVR, dyn s cm^−^^5^	654.1 ± 264.1	119.3 ± 38.8	<0.001	593.5 ± 240.0	116.5 ± 32.5	<0.001	687.7 ± 263.9	132.0 ± 24.6	<0.001	667.6 ± 275.4	101.9 ± 71.4	<0.001
Cardiac output, l min^−1^	5.3 ± 1.9	5.6 ± 0.0	<0.01	5.4 ± 2.0	5.5 ± 0	0.56	5.3 ± 1.2	5.6 ± 0	0.06	5.3 ± 2.0	6.2 ± 2.1	<0.001
Cardiac index, l min^−1^ m^−^^2^	2.9 ± 1.9	3.4 + 0.0	<0.001	3.1 ± 1.0	3.4 ± 0	<0.001	2.9 ± 1.2	3.5 ± 0	<0.001	2.9 ± 1.0	3.4 ± 1.4	<0.001
TRV, m s^−1^	4.0 ± 1.9	2.0 ± 0.0	<0.001	4.0 ± 1.0	1.8 ± 14.1	<0.001	3.9 ± 1.2	2.1 ± 0	<0.001	4.1 ± 1.0	2.7 ± 0.7	<0.001
NT-proBNP, pg ml^−1^	672.7 ± 1279.7	85.4 ± 83.4	<0.001	954.1 ± 1718.0	82.5 ± 41.0	<0.001	505.0 ± 744.2	75.9 ± 36.9	<0.001	626.4 ± 1310.5	292.3 ± 293.5	<0.001
eGFR, ml min^−1^ 1.73 m^−^^2^	83.5 ± 25.9	NR	–	80.3 ± 25.0	NR	–	84.7 ± 24.9	NR	–	84.9 ± 27.1	NR	-
FEV_1_, percent predicted	77.1 ± 20.3	92.1 ± 40.7	<0.001	76.1 ± 19.0	NR	–	76.2 ± 20.1	NR	–	79.2 ± 22.1	92.1 ± 15.0	<0.01
TLC, percent predicted	94.0 ± 12.9	96.2 ± 31.0	0.53	93.9 ± 14.0	NR	–	94.0 ± 13.0	NR	–	94.1 ± 12.1	96.2 ± 11.4	0.61
*D*_LCO_, percent predicted	57.3 ± 24.0	78.3 ± 36.8	<0.001	61.1 ± 25.0	NR	–	55.6 ± 23.7	NR	–	55.9 ± 24.1	78.3 ± 13.6	<0.001

The *P* values were determined by independent two-sample Student’s *t*-test or *χ*^2^ tests. The values are expressed as mean ± s.d.

^a^Individuals treated in outpatient IPAH clinics,

^b^Recruited healthy volunteers,

^c^Individuals with IPAH treated in the intensive care unit,

^d^Non-PH individuals being treated in the intensive care unit,

^e^Blood samples were taken at the time of RHC.

BMI, body mass index; *D*_LCO_, diffusing capacity of the lungs for carbon monoxide; eGFR, estimated glomerular filtration rate; FEV_1_, forced expiratory volume in 1 s; NR, not reported; TLC, total lung capacity.

### Confirmatory western blotting distinguishes individuals with IPAH from those without PH

**Extended Data Fig. 3:**
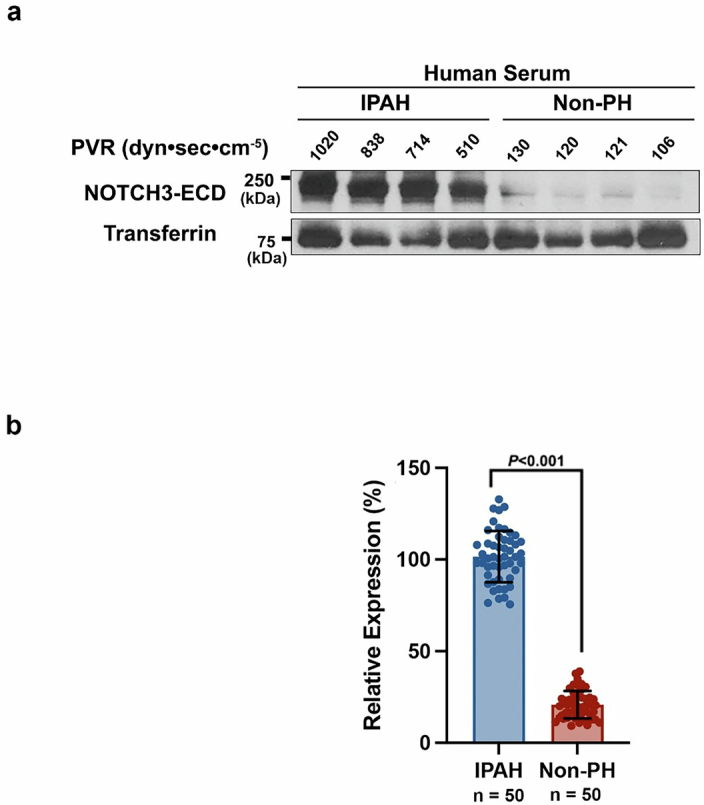
Western blot analysis of levels of serum NOTCH3-ECD in individuals with IPAH compared to levels in individuals without PH. **a**, Representative Western blot analysis of NOTCH3-ECD in the serum of individuals with IPAH compared to individuals without PH. **b**, Relative expression values obtained by densitometry of NOTCH3-ECD relative to transferrin for individuals with IPAH (n = 50) and individuals without PH (n = 50). The *P* value in **b** was determined by independent two-sample t-test. Each blue dot represents a single individual with IPAH, and each red dot indicates an individual without PH in **b**. The data in **b** are expressed as mean ± s.d. PVR = pulmonary vascular resistance.

Blinded validation using western blotting to quantify NOTCH3-ECD confirmed ELISA results, showing higher levels of NOTCH3-ECD in IPAH sera compared to non-PH sera (Extended Data Fig. 3a). Western blot bands normalized to transferrin verified a statistically significant difference between serum NOTCH3-ECD levels in individuals with IPAH compared to individuals without PH (*P* < 0.001) (Extended Data Fig. 3b).

### ELISA specificity for NOTCH3-ECD

**Extended Data Fig. 4:**
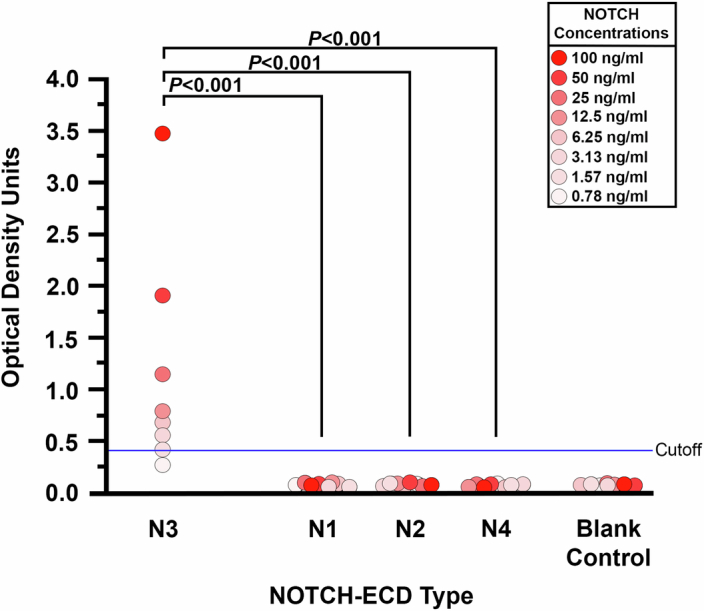
Anti-NOTCH3-ECD antibody is specific for NOTCH3-ECD. ELISA specificity testing of anti-NOTCH3-ECD antibody binding to NOTCH3-ECD but not NOTCH1-ECD, NOTCH2-ECD, and NOTCH4-ECD over the range of 0.78 – 100 ng/ml (*P* < 0.001 for all comparisons). The two-sided *P* values were determined by analysis of variance (ANOVA) with post-hoc Tukey tests. Cutoff = minimal detectable concentration in the assay as defined by the manufacturer. N = NOTCH.

To evaluate the specificity of the anti-NOTCH3-ECD antibody used in ELISA testing, we examined NOTCH1-ECD, NOTCH2-ECD and NOTCH4-ECD over a wide range of concentrations in the assay. The optical density unit values for NOTCH1-ECD, NOTCH2-ECD and NOTCH4-ECD were equal to blank controls over concentrations of 0.78–100 ng ml^−1^, indicating no cross-reactivity when compared to NOTCH3-ECD (*P* < 0.001) (Extended Data Fig. 4).

### NOTCH3-ECD originates from the lung

**Extended Data Fig. 5:**
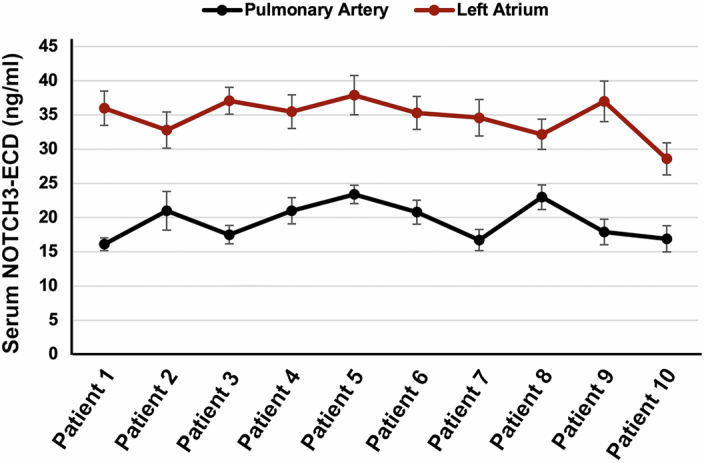
NOTCH3-ECD originates from the lung. Serum NOTCH3-ECD levels, quantified by ELISA, in the left atrium compared to the left or right main pulmonary arteries in individuals with IPAH who underwent cardiac catheterization (n = 10). The data are expressed as mean ± s.d.

NOTCH3-ECD levels were significantly higher in left atrial serum compared to serum from the left or right main pulmonary artery in patients with IPAH, suggesting that the pulmonary vasculature is the source of NOTCH3-ECD (Extended Data Fig. 5), where NOTCH3 is known to be highly expressed^7^.

### Blood test accurately diagnoses IPAH

**Fig. 2:**
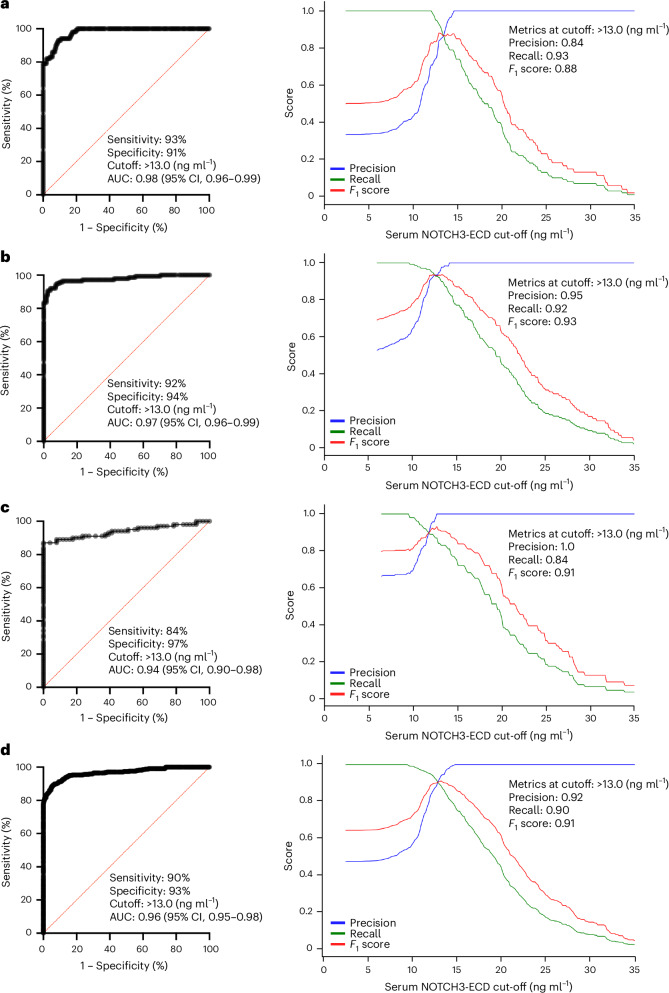
NOTCH3-ECD serum levels effectively discriminate between individuals with IPAH and individuals without PH. **a**–**d**, For the San Diego (**a**), Phoenix (**b**), Boston (**c**) and combined study cohort (**d**), receiver operating characteristic (ROC) curves (left) and *F*_1_-recall-precision plots (right) for diagnosing individuals with IPAH according to serum levels of NOTCH3-ECD. Logistic regression was used to generate ROC curves. AUC, area under the ROC curve with 95% CIs.

Serum NOTCH3-ECD levels effectively discriminated between individuals with IPAH and healthy controls in the individual and combined geographical cohorts. The optimal cutoff value for maximum diagnostic sensitivity and specificity was determined within the San Diego cohort initially at 13.0 ng ml^−1^ (area under the curve (AUC): San Diego: 0.98 (95% confidence interval (CI), 0.96–0.99); sensitivity: 93%; specificity: 91%; precision: 0.84; recall: 0.93; and *F*_1_ score: 0.88) (Fig. 2a). Then, the cutoff of 13.0 ng ml^−1^ was externally applied to and validated in the Phoenix cohort: (AUC: 0.97 (95% CI, 0.96–0.99); sensitivity: 92%, specificity: 94%, precision: 0.95, recall: 0.92; and *F*_1_ score: 0.93) (Fig. 2b) and the Boston cohort (AUC: 0.94 (95% CI, 0.90—0.98); sensitivity: 84%; specificity: 97%; precision: 1.0; recall: 0.84; and *F*_1_ score: 0.91) (Fig. 2c) independently. For the combined cohort, the AUC was 0.96 (95% CI, 0.95–0.98) with sensitivity: 90%, specificity: 93%, precision: 0.92, recall: 0.90; and *F*_1_ score: 0.91 at a cutoff of 13.0 ng ml^−1^ for serum NOTCH3-ECD (Fig. 2d).

**Extended Data Fig. 6:**
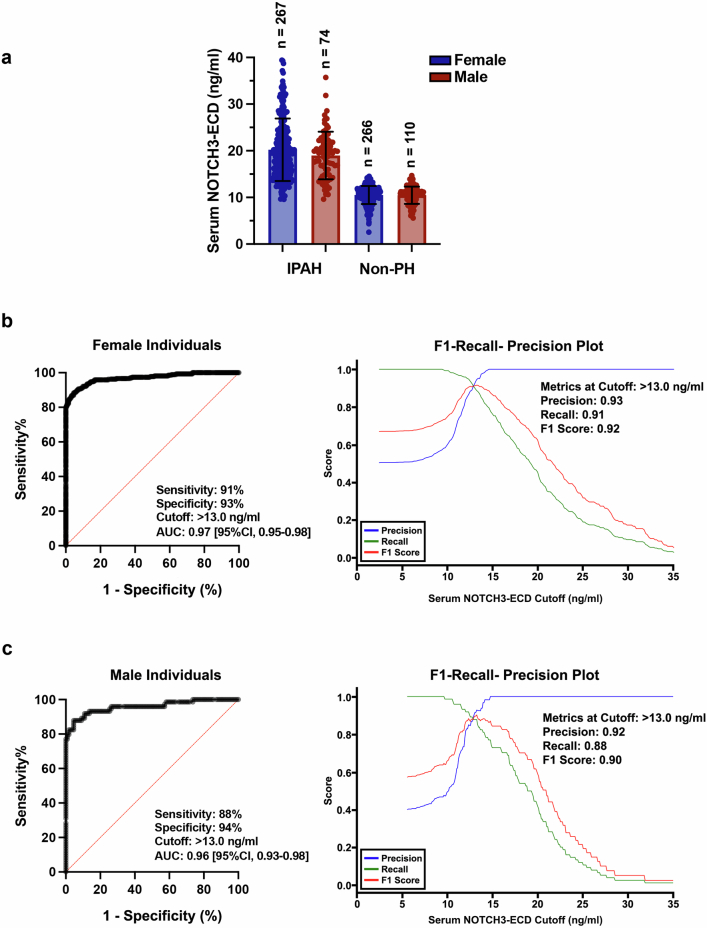
Serum NOTCH3-ECD levels discriminate between individuals with IPAH and without PH for both male and female sexes. **a**, Serum NOTCH3-ECD levels in males and females with IPAH (*P* = 0.14) compared to males and females without PH (*P* = 0.80) in the cross-sectional combined cohort. Each dot represents a single individual. IPAH: n = 74 (males), n = 267 (females); non-PH: n = 110 (males), n = 266 (females). **b, c**, left panels, receiver operating characteristics (ROC) curves for diagnosis of IPAH in female (**b**) and male (**c**) individuals according to serum NOTCH3-ECD levels. Right panels in **b, c** show F1-recall-precision plots for female (**b**) and male (**c**) cohorts. Logistic regression was used to generate ROC curves. AUC: area under the ROC curve with 95% confidence intervals (CI) (**b, c**). The two-sided *P* values above were determined by independent two-sample t-test. The data in **a** are expressed as mean ± s.d.

**Extended Data Fig. 7:**
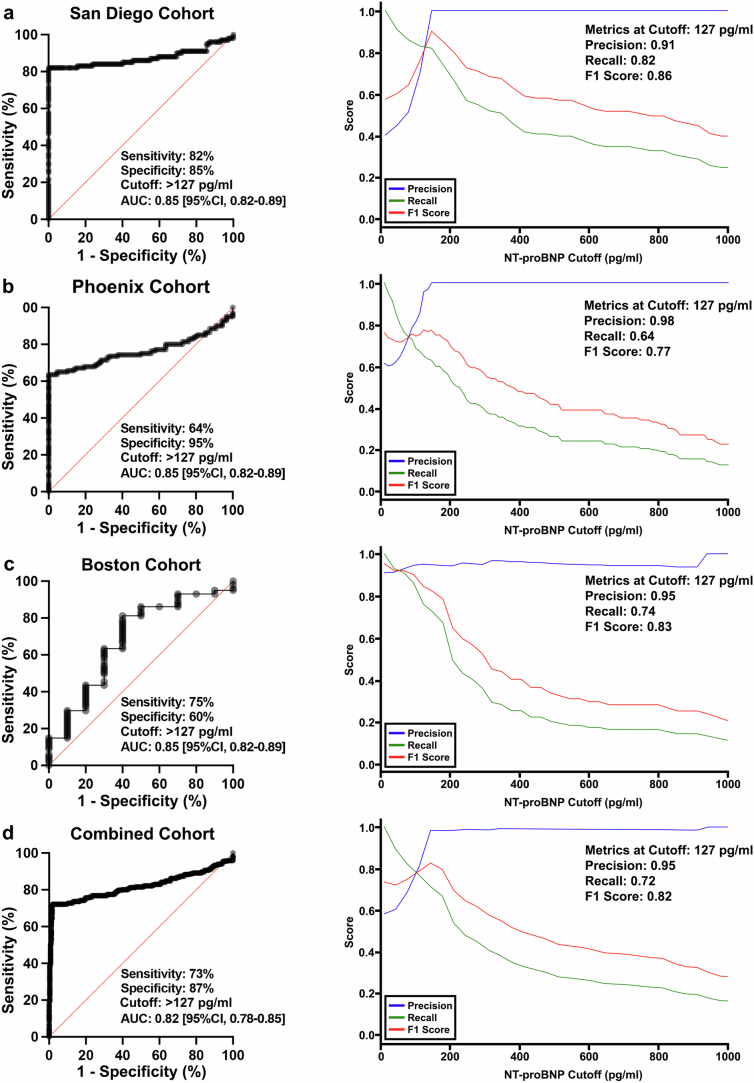
NT-proBNP serum levels in individuals with IPAH and individuals without PH. **a-d**, left panels, receiver operating characteristics (ROC) curves per study cohort: San Diego (**a**), Phoenix (**b**), and Boston (**c**), and the cross-sectional combined cohort (**d**) for diagnosing individuals with IPAH according to serum levels of NT-proBNP. Right panels in **a-d** show F1-recall-precision plots for each of the cohorts. Logistic regression was used to generate ROC curves. AUC: area under the ROC curve with 95% confidence intervals (CI) (**a-d**, left panels).

Serum NOTCH3-ECD distinguished IPAH from non-PH for both women and men in the cross-sectional combined cohort (Extended Data Fig. 6a), with similar efficacy (women: AUC: 0.97 (95% CI, 0.95–0.98); men: AUC: 0.96 (95% CI, 0.93–0.98)) (Extended Data Fig. 6b,c). The diagnostic value of serum NOTCH3-ECD was more robust than serum NT-proBNP in all three geographical cohorts (San Diego, Phoenix and Boston) as well as the cross-sectional combined cohort (Extended Data Fig. 7a–d).

### Elevated serum NOTCH3-ECD is specific to IPAH

**Fig. 3:**
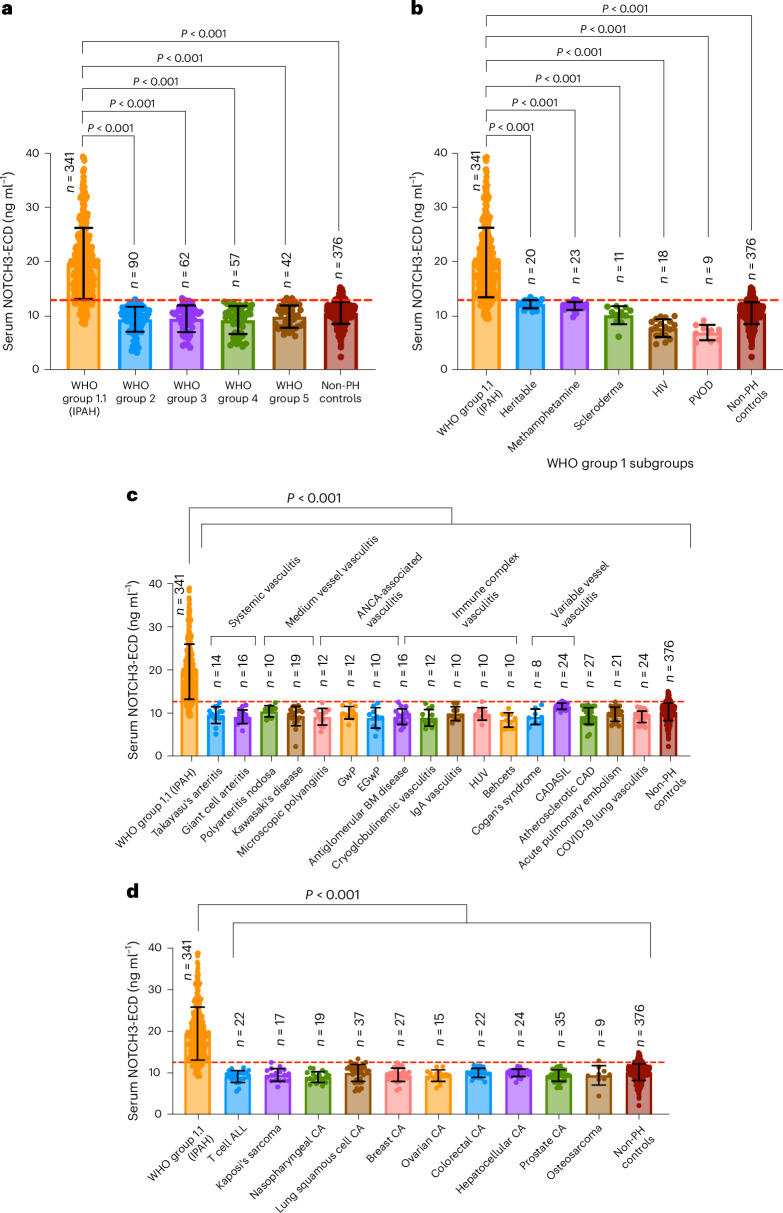
Elevated serum NOTCH3-ECD levels are specific to WHO group 1.1 PAH (IPAH). **a**, Comparison of NOTCH3-ECD serum levels in individuals with WHO group 1.1 (IPAH), individuals with WHO groups 2–5 PH and control individuals without PH (*P* < 0.001 for each group). **b**, Comparison of serum NOTCH3-ECD levels in individuals with WHO group 1 PAH subgroups and control individuals without PH (*P* < 0.001 for each group). **c**, Comparison of serum NOTCH3-ECD levels in individuals with WHO group 1.1 (IPAH), individuals with non-PH vascular diseases and control individuals without PH (*P* < 0.001 for each group*)*. **d**, Comparison of serum NOTCH3-ECD levels in individuals with WHO group 1.1 (IPAH), individuals with NOTCH3-expressing malignancies and control individuals without PH (*P* < 0.001 for each group). The two-sided *P* values in **a**–**d** were determined by ANOVA and post-hoc Tukey tests. Each dot represents a single individual. The red line in **a**–**d** represents a cutoff of 13.0 ng ml^−1^. The data in **a**–**d** are expressed as mean ± s.d. ALL, acute lymphoblastic leukemia; ANCA, anti-neutrophil cytoplasmic antibody; BM, base membrane; CA, carcinoma; CAD, coronary artery disease; CADASIL, cerebral autosomal dominant arteriopathy with subcortical infarcts and leukoencephalopathy; EGwP, eosinophilic granulomatosis with polyangiitis; GwP, granulomatosis with polyangitiis; HIV, human immunodeficiency virus; HUV, hypocomplementemic urticarial vasculitis; PVOD, pulmonary veno-occlusive disease.

In secondary analysis, serum NOTCH3-ECD levels from individuals with IPAH from the combined cohort (San Diego, Phoenix and Boston) were significantly higher than in an external cohort of individuals with WHO groups 2–5 PH (Fig. 3a). In addition, elevations of serum NOTCH3-ECD were not observed in individuals with other WHO group 1 subgroups, including heritable PAH (Fig. 3b), other vasculitides including cerebral autosomal dominant arteriopathy with subcortical infarcts and leukoencephalopathy (CADASIL) (Fig. 3c), or malignancies known to express NOTCH3 (Fig. 3d). There was no significant difference in serum NOTCH3-ECD levels for controls without PH compared to individuals with WHO group 1 subgroups other than 1.1, WHO groups 2–5, vasculitides or malignancies known to express NOTCH3 (all *P* > 0.05) (Fig. 3a–d).

### Serum NOTCH3-ECD levels predict 3-year survival of individuals with IPAH

**Fig. 4:**
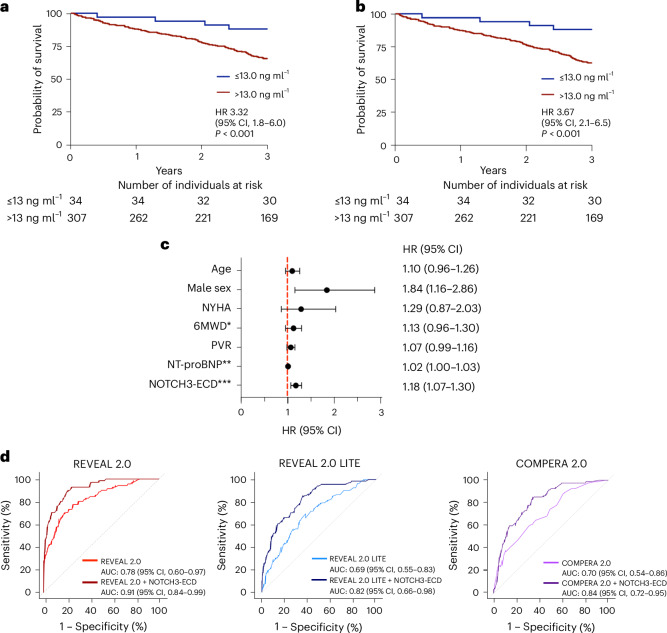
Elevated serum NOTCH3-ECD levels are associated with increased mortality in individuals with IPAH and measurement of serum NOTCH3-ECD levels improves the predictive ability of current PAH prognostic calculators. **a**, Kaplan–Meier plot estimates of overall survival for individuals with IPAH whose serum NOTCH3-ECD levels were higher than the diagnostic cutoff (13.0 ng ml^−1^) compared to individuals with IPAH whose serum NOTCH3-ECD levels were below the cutoff (*P* < 0.001). **b**, Kaplan–Meier plot estimates of transplant-free survival for individuals with IPAH whose serum NOTCH3-ECD levels were higher than the diagnostic cutoff (13.0 ng ml^−1^) compared to individuals with IPAH whose serum NOTCH3-ECD levels were below the cutoff (*P* < 0.001). A log-rank test was used to compare individuals with serum NOTCH3-ECD levels above and below the predetermined cutoff value. **c**, HRs were determined by time-dependent Cox’s regression analysis, for 3-year all-cause mortality risk, with adjustment for patient age, sex, NYHA class, 6MWD, PVR, NT-proBNP and NOTCH3-ECD (*n* = 341). Each dot represents a point estimate of the HR. The vertical red line represents an HR of 1, which is indicative of no effect. ^*^HR per 100 m of 6MWD, ^**^HR per 100 pg ml^−1^ of NT-proBNP and ^***^HR per 3 ng ml^−1^ of serum NOTCH3-ECD >13.0 ng ml^−1^. Data are presented as HRs with 95% CIs. HRs in bold indicate statistically significant differences (*P* < 0.05). **d**, The effect of adding serum NOTCH3-ECD to prognostic calculators, REVEAL 2.0, REVEAL 2.0 Lite and COMPERA 2.0, with machine learning (XGBoost) on 3-year mortality risk prediction in individuals with IPAH. Logistic regression was used to generate ROC curves. AUC, area under the curve with 95% CIs.

Kaplan–Meier survival curves for all 341 individuals with IPAH in the combined cohort (San Diego, Phoenix and Boston) demonstrated that those with serum NOTCH3-ECD levels above the diagnostic cutoff of 13.0 ng ml^−1^ were 3.3× more likely to die within 3 years (hazard ratio (HR) 3.32, 95% CI, 1.8–6.0; *P* < 0.001) (Fig. 4a) and 3.7× more likely to die or undergo a transplantation within 3 years (HR 3.67, 95% CI, 2.1–6.5; *P* < 0.001) (Fig. 4b). During the 3-year follow-up, 102 deaths occurred. There were 36, 29 and 37 deaths at 1 year, 2 years and 3 years, respectively. Seven, 11 and 12 patients (total *n* = 30) were lost to follow-up within 1 year, 2 years and 3 years of sample collection, respectively. In addition, 10 individuals received lung and/or heart–lung transplants within 3 years of follow-up, at days 33, 219, 496, 539, 672, 775, 943, 971, 982 and 1,028.

In addition, after adjustment for patient age, sex and major IPAH prognostic factors (NYHA class, 6MWD, PVR and NT-proBNP) and NOTCH3-ECD in Cox’s multivariate regression, the risk of 3-year mortality rose by 18% per 3 ng ml^−1^ of serum NOTCH3-ECD above the diagnostic cutoff of 13.0 ng ml^−1^ (HR 1.18 (95% CI, 1.07–1.30); *P* < 0.001) (Fig. 4c).

### Serum NOTCH3-ECD levels improve the predictive ability of PAH risk calculators

Using the XGBoost machine learning algorithm, we determined that the addition of serum NOTCH3-ECD levels improved the predictive ability of several established PAH risk calculators. Specifically, the AUC for REVEAL 2.0 improved from 0.78 (95% CI, 0.60–0.97) to 0.91 (95% CI, 0.84–0.99), REVEAL 2.0 Lite rose from 0.69 (95% CI, 0.55–0.83) to 0.82 (95% CI, 0.66–0.98) and COMPERA 2.0 increased from 0.70 (95% CI, 0.54–0.86) to 0.84 (95% CI, 0.72–0.95) (Fig. 4d). Additional model metrics are displayed in Extended Data Table 1.

### Serum NOTCH3-ECD level predicts IPAH in treatment-naive individuals and correlates with disease progression

**Fig. 5:**
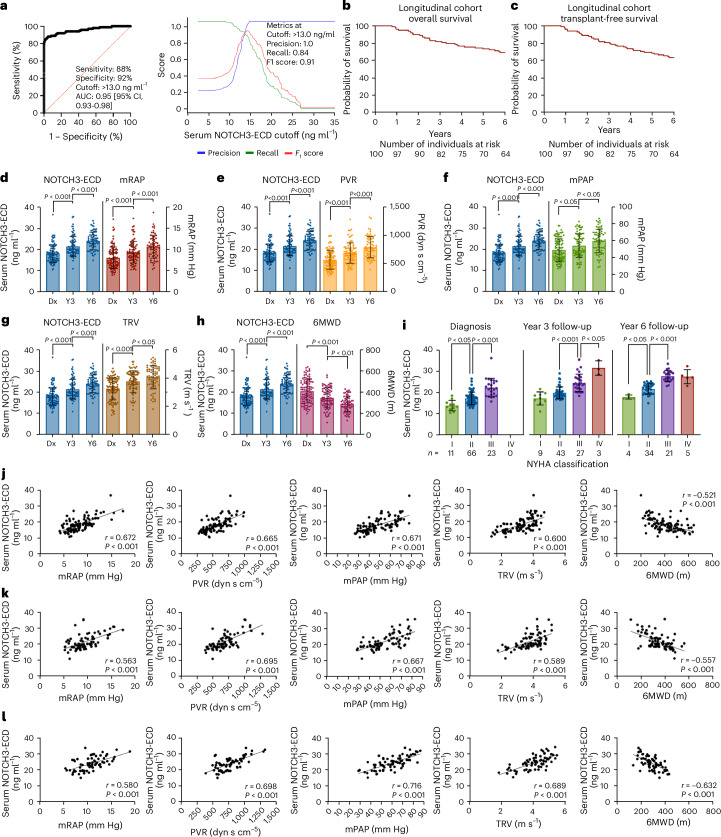
Elevated serum NOTCH3-ECD levels diagnose treatment-naive individuals with IPAH and correlate with IPAH disease parameters during a 6-year longitudinal follow-up. **a**, ROC curve for diagnosis of treatment-naive individuals with IPAH according to serum NOTCH3-ECD levels (left) and *F*_1_-recall-precision plot (right). **b**, Kaplan–Meier plot estimates of overall survival of 100 treatment-naive individuals with IPAH followed over 6 years (*n* = 100 at diagnosis, *n* = 82 at 3 years and *n* = 64 at 6 years). **c**, Kaplan–Meier plot estimates of transplant-free survival of the same longitudinal cohort. **d**–**h**, Comparison of serum NOTCH3-ECD levels with disease progression over 6 years of follow-up, as measured by mRAP (*P* < 0.001) (**d**), PVR (*P* < 0.001) (**e**), mPAP (*P* < 0.05) (**f**), TRV (*P* < 0.001 for TRV between diagnosis and year 3; *P* < 0.05 for TRV between year 3 and year 6) (**g**) and 6MWD (*P* < 0.001 for 6MWD between diagnosis and year 3; *P* < 0.01 for 6MWD between year 3 and year 6) (**h**) (*n* = 100 at diagnosis, *n* = 82 at 3 years and *n* = 64 at 6 years). **i**, Comparison of serum NOTCH3-ECD levels with disease progression as measured by NYHA class over 6 years of follow-up (*P* < 0.05 or *P* < 0.001 as indicated) (*n* = 100 at diagnosis, *n* = 82 at 3 years and *n* = 64 at 6 years). Each dot represents a single individual. **j**–**l**, Correlations between serum NOTCH3-ECD levels with parameters: mRAP, PVR, mPAP, TRV, and 6MWD at diagnosis (**j**), at year 3 of follow-up (**k**) and at year 6 of follow-up (**l**) in treatment-naive individuals with IPAH. The *P* values in **j**–**l** were <0.001. Logistic regression in **a** was used to generate ROC curves. The two-sided *P* values in **d**–**i** were determined by ANOVA with a post-hoc Tukey test. For dot plots in **j**–**l**, *r* and *P* values were calculated using Spearman’s rank correlation. The data in **d**–**i** are expressed as mean ± s.d. Dx, diagnosis; Y3, year 3; Y6, year 6.

A separate longitudinal cohort of 100 newly diagnosed, treatment-naive IPAH individuals was followed over 6 years, with a median follow-up time of 6.69 years (IQR, 3.82–6.87 years). The diagnostic cutoff of 13.0 ng ml^−1^ for serum NOTCH3-ECD was applied to the treatment-naive IPAH individuals and demonstrated an AUC of 0.95 (95% CI, 0.93–0.98) with sensitivity 88%, specificity 92%, precision 1.0, recall 0.84 and *F*_1_ score 0.91 (Fig. 5a). There were 17 and 30 deaths at 3 years and 6 years, respectively (Fig. 5b). Six patients received a heart and/or lung transplantation, at days 398, 1,112, 1,235, 1,501, 1,629 and 1,860 after diagnosis, within the course of the study (Fig. 5c). No living patients were lost to follow-up and individuals in the longitudinal cohort were not genotyped. Serum NOTCH3-ECD predicted the presence and progression of disease as measured by mRAP, PVR, mPAP, TRV, 6MWD (Fig. 5d–h) and NYHA class (Fig. 5i). The mean serum NOTCH3-ECD level rose by 4.21 ± 2.7 ng ml^−1^ at 3 years (*P* < 0.001) and 6.24 ± 4.0 ng ml^−1^ at 6 years (*P* < 0.001) compared to baseline at the time of diagnosis. The rise in serum NOTCH3-ECD correlated with significant increases in mRAP, PVR, mPAP and TRV and decreases in 6MWD at 3 years (mean change: mRAP 1.3 ± 3.6 mm Hg (*P* < 0.001), PVR 140 ± 133.1 dyn s cm^−5^ (*P* < 0.001), mPAP 6.8 ± 7.2 mm Hg (*P* < 0.05), TRV 0.7 ± 0.9 m s^−1^ (*P* < 0.001) and 6MWD −62.1 ± 126.8 m (*P* < 0.001)) and at 6 years (mean change: mRAP 2.7 ± 4.0 mm Hg (*P* < 0.001), PVR 259 ± 159.2 dyn s cm^−5^ (*P* < 0.001), mPAP 13.7 ± 9.6 mm Hg (*P* < 0.05), TRV 1.1 ± 0.8 m s^−1^ (*P* < 0.05) and 6MWD −117.9 ± 51.2 m (*P* < 0.01)) (Fig. 5d–h,j–l).

**Extended Data Fig. 8:**
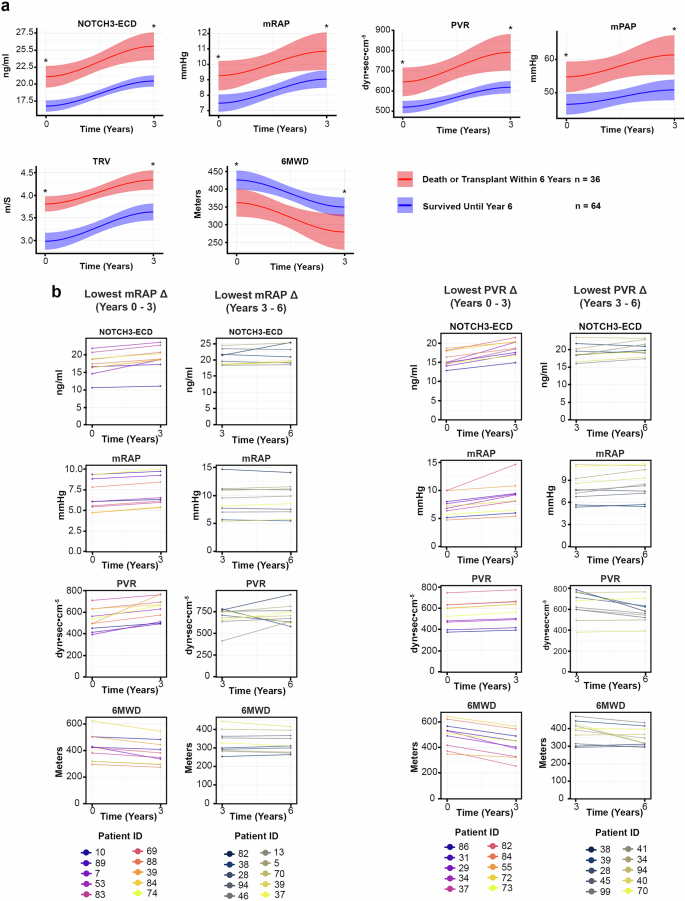
Serum NOTCH3-ECD parallels disease progression in individuals with rapidly worsening disease leading to transplant or death, as well as those with periods of clinically stable disease. **a**, Locally estimated scatterplot smoothed (LOESS) mean longitudinal trends of serum NOTCH3-ECD levels, mRAP, PVR, mPAP, TRV, and 6MWD, over the first three years following enrollment. Patients were stratified by clinical outcome: those who died or underwent transplantation within six years (red, n = 36) and those who survived to year six (blue, n = 64). *In bold indicates statistical significance between both groups at each time point (*P* < 0.05). Solid lines represent LOESS mean trend. Shaded areas demonstrate the 95% confidence interval (CI) for the mean trend. LOESS plots were generated using the ggplot2 package in R. **b**, Individual longitudinal trajectories of serum NOTCH3-ECD levels, mRAP, PVR, and 6MWD over early (Years 0 – 3, left panels) and late (Years 3 – 6, right panels) disease intervals in patients with IPAH with the lowest change (Δ) in either mRAP (left two columns) or PVR (right two columns). Each line represents an individual patient, color-coded by ID across variables with each time interval.

Patients who ultimately developed worsening disease resulting in death or transplantation within 6 years presented with a higher initial burden of disease with significantly elevated serum NOTCH3-ECD, mRAP, PVR, mPAP and TRV, as well as a lower 6MWD, compared to those who survived until the end of the study (*P* < 0.05 for all variables at baseline) (Extended Data Fig. 8a). Using mixed analysis of variance (ANOVA), we confirmed significant clinical worsening over time (diagnosis to year 3) and significant differences between groups over the 3 years for each clinical variable (mRAP, PVR, mPAP, TRV and 6MWD) and serum NOTCH3-ECD levels (*P* < 0.05 for all variables for time and group analyses). These results indicate that, not only were baseline values worse in the death or transplantation group, but also the trend of clinical deterioration continued, during which hemodynamic parameters and serum NOTCH3-ECD worsened and remained significantly greater than that of survivors by year 3 of follow-up (*P* < 0.05 for all variables at year 3).

Although many patients demonstrated progression of disease during the longitudinal study, some individuals had interval periods of stable disease. To better understand these trends, we identified patients with the lowest clinical changes in mRAP or PVR over two 3-year periods. Specifically, we followed the hemodynamic and serum NOTCH3-ECD trends for ten patients from diagnosis to year 3 and then another ten individuals from year 3 to year 6. During these periods of disease quiescence, individual serum NOTCH3-ECD levels stabilized, corresponding to minimal changes seen in mRAP, PVR and 6MWD (Extended Data Fig. 8b).

## Discussion

The identification of a quantitative blood biomarker that can uniquely diagnose WHO group 1 IPAH, correlate with disease severity and predict risk of death has potential therapeutic application for individuals with this disease. This study demonstrates the following: (1) NOTCH3-ECD, a cleavage product of NOTCH3 signaling in sPASMCs, is shed into the circulation and is detectable using a simple, reproducible assay; (2) NOTCH3-ECD can accurately predict the presence of IPAH in treatment-naive and treated individuals and serum levels of this peptide correlate with disease severity; (3) serum NOTCH3-ECD can independently predict 3-year survival in individuals with IPAH; (4) serum NOTCH3-ECD improves established prognostic PAH models; (5) serum NOTCH3-ECD as a biomarker is equally efficacious in women and men; and (6) serum NOTCH3-ECD is specific for WHO group 1.1 IPAH.

Defining the mechanistic roles of the NOTCH3-ECD and NOTCH3-ICD may be critical to the design of biomarkers and successful therapies for IPAH. NOTCH3-ICD, resulting from cleavage of the NOTCH3 receptor, is known to modulate the transcription factor, HES-5, which is required for vSMC proliferation and anti-apoptosis, and self-renewal of SMC progenitors^12–14^. Previous studies have shown correlation between the amount of NOTCH3-ICD and HES-5 in sPASMCs and in the medial layer of pre-capillary pulmonary arteries of individuals with IPAH with worsening PVR^7^. Inhibition of NOTCH3 cleavage, resulting in diminished NOTCH3-ICD, using pharmacological blockade^7,15^, anti-drug conjugates^16^ or monoclonal antibodies^10^, has been shown to reverse PH in rodent and pig disease models. Although NOTCH3-ICD has been extensively studied in the lung vasculature, little is known about the fate of the other NOTCH3 cleavage product: NOTCH3-ECD.

Several lines of evidence have shown that Notch cleavage occurs when the membrane-bound Notch ligand binds to the ECD and mechanically pulls it toward the ligand-presenting cell, opening up the cleavage site for Notch receptor proteolysis^17^. After Notch cleavage, the ligand is endocytosed into the ligand-presenting cell, which may be the receptor-expressing cell itself (*cis*-activation or autocrine activation) or the adjacent cell (*trans*-activation or paracrine activation) and recycled for presentation on the cell surface^10,17,18^. Studies in *Drosophila* and mammalian cell lines have suggested that, after cleavage of the Notch receptor, Notch-ECD is liberated from the plasma membrane and released into the interstitium and blood, whereas the Notch ligand (Jag-1) is internalized into the ligand-presenting cell^19^. In other circumstances, the entire Notch-ECD/Jag-1 complex may be endocytosed and internalized into the ligand-presenting cell, with Jag-1 recycled back to the cell surface and Notch-ECD degraded^17^.

Building on this work, we have shown that the highest serum levels of NOTCH3-ECD in patients with IPAH are found in the left atrium compared to the right and left main pulmonary arteries, suggesting that NOTCH3-ECD originates in the pulmonary vasculature. It is interesting that, although serum NOTCH3-ECD can accurately diagnose IPAH and protein levels correlate with IPAH severity over time, we found no difference in NOTCH3-ECD levels in relation to drug regimen. This is likely due to the disparity in drug treatment regimens and dosages with respect to disease severity in the three different centers where patients were accrued. For example, one center used mostly a three-drug regimen for patients with modest elevation of PVR, whereas others used a one-drug or two-drug regimen for the same severity of disease.

To date, there is no lung-specific noninvasive test used clinically to diagnose and predict the presence and severity of IPAH. Although blood tests, such as immune-associated signatures^20^, inflammatory cluster proteins^21,22^, endothelial microvesicles^23^, select microRNAs^24–26^ and nonselective biomarkers^6,27–31^, have been studied in PAH, none has widespread clinical use in a cost-effective manner. Functional assessment with cardiac magnetic resonance imaging^32^ and magnetic resonance elastography^33^ is promising, but is costly, lacks specificity for a single PH WHO group and is not widely available. Although risk stratification calculators (REVEAL^34^, REVEAL 2.0^35^, REVEAL 2.0 Lite^35^, COMPERA 2.0^36^, European Society of Cardiology or European Respiratory Society guidelines^2^ and French noninvasive criteria^37^) employ clinical data that are scored to predict PH outcome, they use differently weighted parameters and can be improved. Here we demonstrate that NOTCH3-ECD can be utilized independently to monitor disease progression and prognosis, as well as improve previously validated mortality risk models. Tests used to diagnose and follow patients with IPAH are limited by expense, radiation exposure and patient discomfort and do not always rule out other WHO group PH diseases. Markers of cardiac strain (NT-proBNP and BNP) have been utilized in the diagnosis of PAH, as well as serving as a metric of prognosis and progression of disease, but are not specific to one WHO group^38^. Specifically, NT-proBNP has been found to be useful as a parameter in multi-parameter risk PAH calculators, but less efficacious as a sole diagnostic or prognostic tool^39,40^. The strength of the serum NOTCH3-ECD assay is that it is unique to WHO group 1.1 IPAH and serves as an independent, specific marker for diagnosis and surrogate for disease progression in the pulmonary vasculature.

Recent studies have suggested that there is considerable heterogeneity in the clinical presentation and biological parameters in different forms of PAH and PH. Several investigative studies have addressed differential responses to provocative maneuvers during cardiac catheterization^41,42^ and variation in cardiac magnetic resonance imaging parameters^43–45^. These functional and imaging variables often reflect underlying differences in vascular remodeling and right ventricular adaptation, pointing to diverse pathophysiological trajectories. Indeed, the clinical landscape of IPAH is further complicated by associations with conditions like sleep apnea and sleep-related hypoxia^46^, low *D*_LCO_^47^, obesity^48^ and the occurrence of atrial fibrillation^48^ and heart failure with preserved ejection fraction^49^, each of which can modulate disease phenotype and potentially add to IPAH-specific signaling pathways. In addition, molecular mechanisms have been shown to mirror the clinical heterogeneity with distinct clustering in plasma proteomics^50,51^, varied peripheral blood mononuclear cell fraction immunophenotyping^52^ and whole-blood transcriptomic profiling^53^. Collectively, these findings suggest that IPAH is not a uniform disease, as further demonstrated by perturbations in metabolomics^54,55^, lipid ratios^56^ and mitochondrial dysfunction^57^. Although we recognize that IPAH may have heterogeneous clinical and biological profiles, our data suggest that serum NOTCH3-ECD may serve as a unifying diagnostic and prognostic marker for IPAH. Integration of serum NOTCH3-ECD testing into the clinical workup of patients with WHO group 1.1 disease has the potential to improve the initial identification of this disease and serve as a noninvasive adjunct for disease monitoring.

There are several limitations to this study. First, we utilized a hypothesis-driven approach to examine one specific signaling pathway in the generation of a unique biomarker of IPAH. We recognize that other independent or interfacing signaling cascades and pathways^58^ that interact with NOTCH may also play a role in this disease. Second, the NOTCH3-ECD assay will require additional validation with respect to multiple races and ethnicities over a larger cohort. Third, PAH patients do not routinely undergo genotyping for heritable disease in the United States of America. However, in individuals with heritable PAH with BMPR2 mutations confirmed in at least two generations, serum NOTCH3-ECD was not significantly elevated compared to non-PH individuals, suggesting differences in disease mechanism. Finally, we recognize that a small percentage of patients diagnosed with IPAH may have mutations consistent with heritable PAH. In the future, low serum NOTCH3-ECD levels may serve as a screening tool for identifying the group of individuals with IPAH who should undergo genotyping to determine whether they truly exhibit heritable PAH.

In summary, this study demonstrates that serum NOTCH3-ECD is a sensitive and specific biomarker for IPAH, with the ability to accurately and noninvasively detect disease presence, severity and survival. The diagnostic and prognostic capabilities of this simple blood test are excellent and can be an adjunct to current modalities of hemodynamic measurements, while being broadly accessible and noninvasive. Widespread clinical adoption of NOTCH3-ECD testing has the future potential to transform the IPAH diagnosis and management landscape, enabling possibly earlier treatment and improved outcomes. Further validation in expanded cohorts of individuals and the development of point-of-care assays will maximize clinical utility. The serum NOTCH3-ECD biomarker test represents an advance toward overcoming barriers in effectively screening, monitoring and risk-stratifying individuals with this progressive, fatal disease.

## Methods

### Study populations and sample collection

#### Cross-sectional cohorts

Between 2010 and 2021, serum samples were prospectively collected from 341 individuals with IPAH and 376 without PH (Table 1) being treated at the University of California, San Diego (UCSD) (San Diego cohort: 100 IPAH, 200 non-PH), the University of Arizona (Phoenix cohort: 140 IPAH, 125 non-PH) and the Massachusetts General Hospital (Boston cohort: 101 IPAH, 51 non-PH) (Table 1). A sample size of 341 individuals with IPAH and 376 individuals without PH was calculated to provide 90% power to detect a minimum effect size of 0.27 for the difference in serum NOTCH3-ECD levels between the two groups with a two-sided *α* = 0.05. The San Diego and Phoenix IPAH patient samples were collected on an outpatient basis, whereas the Boston patients with IPAH had serum collected during ICU hospitalization for IPAH. Informed consent was obtained for all individuals from each cohort. Patient sex was determined by assigned sex according to each patient’s hospital records. All IPAH blood samples were collected within 1 month of RHC or ECHO. Control individuals in the San Diego and Phoenix cohorts were healthy paid volunteers, whereas the Boston control cohort comprised ICU patients without PH, being treated for other non-lung-related diseases (51 nonintubated patients comprising 37 multi-trauma orthopedic patients without lung contusion, acute respiratory distress syndrome or pulmonary embolism; 10 individuals recovering from elective large abdominal, vascular or orthopedic operations; 3 patients with a closed head injury; and 1 patient with amyotrophic lateral sclerosis). All individuals, including controls, underwent RHC as part of this study. All IPAH patients tested negative for HIV and active hepatitis viral infection, as well as anti-nuclear, anti-centromere, anti-mitochondrial, anti-double-stranded DNA, anti-topoisomerase 1, anti-Ro and anti-La antibodies. No patient with IPAH was undergoing evaluation for liver transplantation or had received a previous liver transplant. Patients with IPAH did not undergo genetic testing at the time of diagnosis and none had a family history of PAH (WHO group 1) or other PH (WHO groups 2–5). The determination of IPAH was made by an integrated assessment by an experienced PH pulmonologist or cardiologist, with further adjudication as needed by a committee of PH physicians. Inclusion and exclusion criteria for the diagnosis of IPAH is given in Supplementary Table 1.

Patients with subtypes of WHO group 1 PAH associated with previously diagnosed heritable mutations, methamphetamine, scleroderma, HIV, congenital heart defects, portal hypertension or pulmonary veno-occlusive disease were not included in the primary analysis, although serum samples from these patients were collected from UCSD and PH centers in the United States and United Kingdom for secondary comparative analysis. Serum samples were also obtained from individuals with non-PH vasculitides that affect the lung as well as individuals with malignancies expressing NOTCH3 for additional secondary comparative analysis. Informed consent was obtained for all individuals at participating institutions for the above patient groups. Sample numbers for secondary comparative analyses were determined by availability of serum samples over the 9 years of collection from multiple participating institutions, UCSD and the UCSD Biorepository. Only IPAH and non-PH serum samples were included in the primary analysis.

#### Longitudinal cohort

A separate cohort of 100 newly diagnosed, treatment-naive patients with IPAH (43 patients from San Diego, 57 patients from Phoenix), were followed for 6 years with serial blood sampling, ECHO and RHC (Extended Data Table 2). Informed consent was obtained from all 100 individuals. Sex was determined by assigned sex according to each patient’s hospital records. No living patients were lost to follow-up during the time course of the study. Blood samples were taken on the day of RHC at diagnosis, at 3 years and at 6 years. Sample numbers for longitudinal analysis were determined by availability of serum samples collected at three timepoints for each patient over a 6-year period. These patients were analyzed separately from cross-sectional cohorts.

#### Sample collection

Blood samples were collected by venipuncture in heparin-coated tubes (Becton Dickinson) within 1 month of the most recent RHC or ECHO. Serum was separated by Ficoll (Amersham) gradient centrifugation of whole blood and was stored at −80 °C. Serum samples were de-identified using a barcode at the point of care before being transported to the lab for analysis. For ten patients, serum was derived from blood sampled from the pulmonary artery and transseptally from the left atrium during cardiac catheterization to investigate the origin of NOTCH3-ECD as part of institutional review board (IRB)-approved research protocols. All studies were approved by the UCSD Human Subjects Program and relevant IRB committees of all participating institutions, with the following protocols: UCSD: IRB protocol no. 809539; Mass General Brigham: IRB protocol no. 2010P000982; University of Arizona: IRB protocol no. 1100000621A013, University of Alabama: IRB protocol no. 1639383-7; University of New Mexico: IRB protocol no. 00003255; University of California, Los Angeles (UCLA): IRB protocol no. 12-0738; Americas Hospital, Guadalajara, Mexico: IRB protocol no. AH-IRB-B2; Mount Sinai, New York: IRB protocol no. RC-4590; and University of Cambridge (UK): IRB and informed consent as part of the UK National Cohort Study of Idiopathic and Heritable Pulmonary Arterial Hypertension (ClinicalTrials.gov ID: NCT01907295; UK Research Ethics Committee reference no. 13/EE/0203).

### ELISA

Levels of NOTCH3-ECD in human serum were quantified in nanograms per milliliter (ng ml^−1^) using the human NOTCH3-ECD ELISA kit (Cloud Clone, cat. no. SEL147Hu) according to the manufacturer’s protocol. Operators who performed the ELISAs were blinded to case and control status of the samples. NOTCH3-ECD standards were reconstituted with 1.0 ml of standard diluent to achieve a high standard of 100 ng ml^−1^. Serial dilution was performed to establish a concentration curve (ng ml^−1^) of 100, 50, 25, 12.5, 6.25, 3.13 1.57 and 0.78 as well as a final blank of 0 ng ml^−1^ Serum samples were diluted 1:5 by mixing 20 μl of serum with 80 μl of phosphate-buffered saline. Each sample of the dilution series, as well as each diluted serum sample (all 100 μl), was placed in triplicate into individual wells of a 96-well anti-NOTCH3-ECD-coated plate and incubated for 1 h at 37 °C. The liquid was removed by decanting each plate. Diluted biotinylated antibody (100 μl) was added to each well. Plates were incubated for 1 h at 37 °C. Wells were covered with 350 μl of the manufacturer’s wash solution and decanted after 1 min. Three wash cycles using 350 μl of the manufacturer’s wash solution were performed. Streptavidin-conjugated horseradish peroxidase, 100 µl, was added to each well. Plates were incubated for 30 min at 37 °C. Five wash cycles with the manufacturer’s wash solution were performed as above. Tetramethylbenzidine substrate solution, 90 µl, was added to each well. Plates were covered with aluminum foil and incubated for 20 min at 37 °C. The reaction was terminated by adding 50 μl of stop solution to each well. Absorbance was measured immediately at 450 nm using a SpectraMax M2e plate reader (Molecular Devices) and the results were collated using SoftMax Pro v5.4 (Molecular Devices). Serum samples were run on different locations on the plate each time that they were assayed, with plates randomly containing IPAH and control samples from each geographical cohort. All experiments were performed in triplicate on 3 days separately using different ELISA lots.

### Cross-reactivity testing

ELISA specificity for the anti-NOTCH3-ECD antibody was tested by adding recombinant human NOTCH1-ECD (Beta Life Sciences, cat. no. BLPSN-3544), NOTCH2-ECD (Beta Life Sciences, cat. no. BLPSN-3547) and NOTCH4-ECD (Beta Life Sciences, cat. no. BLPSN-3549) peptides diluted in phosphate-buffered saline to concentrations of 0.78–100 ng ml^−1^. Cross-reactivity was determined quantitatively by optical density compared to blank controls. Experiments were performed in triplicate on 3 days separately, using different assay lots.

### Immunoprecipitation and western blotting

Immunoprecipitation and western blotting were performed as previously described^59^. The antibodies used for western blotting were: human anti-NOTCH3-ECD antibody (Sigma-Aldrich, clone 2G8, cat. no. MABF937, 1:1,000), goat polyclonal anti-rat secondary antibody (Thermo Fisher Scientific, cat. no. 31470, 1:5,000), rabbit polyclonal anti-transferrin antibody (Thermo Fisher Scientific, cat. no. PA527306, 1:1,000) and horseradish peroxidase-conjugated goat polyclonal anti-rabbit immunoglobulin G antibody (Thermo Fisher Scientific, cat. no. 31460, 1:5,000). For immunoprecipitation experiments, primary antibodies were used at a concentration of 6 μg of antibody per 1 mg of total protein. Experiments were performed in triplicate on 3 days separately.

### Statistical analysis

#### Diagnostic analyses

The primary objective was to assess the ability of serum NOTCH3-ECD to differentiate between individuals with IPAH and individuals without PH. Logistic regression was used to generate ROCs to assess the ability of serum NOTCH3-ECD levels to predict the presence of IPAH in three geographically separate cohorts (San Diego, Phoenix and Boston) and one combined cohort.

Specifically, the optimal cutoff of serum NOTCH3-ECD to maximize diagnostic sensitivity and specificity to differentiate between individuals with IPAH and individuals without PH was determined by calculating the optimal Youden’s Index and *F*_1_ score. The cutoff was established first in the San Diego cohort independently and then externally applied to the Phoenix and Boston cohorts. Finally, the cutoff value was applied to the combined cohort to evaluate potential generalizability and overall performance. The AUC, *F*_1_ score, precision and recall at the defined cutoff were calculated for each cohort independently and then as a combined group.

#### Prognostic analyses

The secondary objective was to assess whether levels of serum NOTCH3-ECD predict mortality in 341 individuals with IPAH (combined cohort from San Diego, Phoenix and Boston) within 3 years of sample collection. Kaplan–Meier plots were constructed with patients censored at the date last known to be alive or the date of lung or heart–lung transplantation. A log-rank test was used to compare individuals with serum NOTCH3-ECD levels above and below the predetermined cutoff values and HRs were estimated using Cox’s proportional hazards models. Separate analysis was also performed for which lung or heart–lung transplantation and mortality were both considered events and the transplant-free survival was calculated. Individual patient mortality was verified from the medical records.

Multivariate time-dependent Cox’s regression was also employed to assess the impact of serum NOTCH3-ECD levels on the 3-year mortality of 341 individuals with IPAH (combined cohort from San Diego, Phoenix and Boston) while adjusting for major prognostic factors for IPAH. The variables included in the final multivariate Cox’s regression model were those with *P* < 0.20 after backward, stepwise, logistic regression predicting 3-year mortality (Supplementary Table 2). The final Cox’s regression model included patient age, sex, NYHA class, 6MWD, PVR, NT-proBNP and serum NOTCH3-ECD.

To compare the ability of the REVEAL 2.0, REVEAL 2.0 Lite and COMPERA 2.0 scores to predict 3-year mortality in IPAH individuals (combined cohort from San Diego, Phoenix and Boston), both with and without the addition of serum NOTCH3-ECD, separate machine learning models were generated (machine learning code in Supplementary Note 1). Specifically, the extreme gradient boost (XGBoost) algorithm was employed due to its effectiveness in handling high-dimension data, robustness and ability to capture complex patterns. In addition, XGBoost is particularly skilled at handling missing data effectively, which typically limits the predictive ability of nomograms and other machine learning tools such as neural networks. As such, no imputation of data was performed or utilized.

The binary classification problem processed using machine learning (survival versus death within 3 years) was implemented using the R caret package. The clinical characteristics of the training set were used as independent variables to develop machine learning models to predict all-cause mortality within 3 years. All variables included in the REVEAL 2.0^35^, REVEAL 2.0 Lite^35^ and COMPERA 2.0^36^ calculators were included in each individual machine learning model, with and without NOTCH3-ECD levels. There were a maximum of 13 categorical variables (demographics: men, age >60 years, eGFR < 60 ml min^−1^ 1.73 m^−^^2^ or renal insufficiency, NYHA class, systolic blood pressure ≥110 mm Hg or <110 mm Hg, heart rate ≤96 beats per min or >96 beats per min, all-cause hospitalizations ≤6 months, 6MWD categories in respect of each calculator, NT-proBNP or BNP values respective to each calculator, pericardial effusion on echocardiogram, percentage predicted *D*_LCO_ ≤ 40, mRAP > 20 mm Hg within 1 year and PVR < 5 Wood units on RHC) and one continuous variable (serum NOTCH3-ECD) included for each model with respect to the corresponding mortality calculator. Data were formatted into a binary framework for each of the categorical variables by converting them into dummy variables. To avoid perfect multicollinearity, the first of the dummy variables was dropped from the model. The final dataset was divided into training (80%) and testing (20%) data subsets.

The models were constructed using the randomForest package in R and hyperparameters, including the number of trees, mtry (number of predictors to sample at each split) and min_*n* (number of observations needed to split nodes). Then, the models were optimized as described previously^60,61^. Briefly, tuning parameters, which are modifiable variables such as the rate of learning, depth and complexity of the model, were tested for each classifier to obtain the best prediction in the training dataset. Bayesian optimization was employed for hyperparameter tuning to iteratively search for the ideal parameters. For XGBoost, the hyperparameters optimized were eta, max_depth, min_child_weight, subsample and nfold. Optimization was performed on the training sub-dataset with the ‘scoring_function’ that evaluated the model based on the resulting AUC.

The risk of mortality was predicted using the test dataset and the predictive performance was evaluated by examining the AUC. The AUC, *F*_1_ score, precision, recall, accuracy and balanced accuracy after *k*-fold crossvalidation were calculated and reported as the mean ± s.d. for all models after tenfold validation. *K*-fold crossvalidation was employed to maximize the utility of available data while providing a robust assessment of model performance. By partitioning the dataset into *k* subsets and iteratively using each subset for validation while training on the remainder, this technique reduces evaluation bias and delivers more reliable performance metrics than traditional single train-test splits.

#### Longitudinal cohort analyses

In a separate, longitudinal cohort of 100 treatment-naive IPAH individuals, the survival, change in serum NOTCH3-ECD levels, mRAP, PVR, mPAP, TRV, 6MWD and NHYA class over 6 years was analyzed. Kaplan–Meier curves were constructed for overall survival as well as transplant-free survival within 6 years of diagnosis. Patients were censored at the date last known to be alive or the date of a lung or heart–lung transplantation.

Trends of serum NOTCH3-ECD levels, mRAP, PVR, mPAP, 6MWD and TRV were compared between patients who developed progressive disease and underwent a transplantation or died within 6 years of follow-up and patients who survived until the end of the study period. To visualize the longitudinal trends of key clinical variables (NOTCH3-ECD, mRAP, PVR, mPAP, TRV and 6MWD) over the study period (years 0, 3 and 6), locally estimated scatterplot smoothing plots were generated using the ggplot2 package in R. Locally estimated scatterplot smoothing is a nonparametric method that fits local polynomial regressions to the data, allowing visualization of the central tendency without assuming a specific global functional form. In these plots, time (in years) was plotted on the *x* axis against the clinical variable’s value on the *y* axis. Each variable was displayed in a separate facet with independent *y*-axis scaling to accommodate differing value ranges. A mixed ANOVA was employed to analyze the role of time and prognostic status (death or transplantation versus survival) on the trends of serum NOTCH3-ECD levels, mRAP, PVR, mPAP, TRV and 6MWD.

Data are presented as mean ± s.d., unless otherwise indicated. Comparison between independent groups for continuous variables was performed using independent two-sample Student’s *t*-test and ANOVA with a post-hoc Tukey test, as indicated. Correlation between continuous variables was assessed by Spearman’s rank correlation coefficient. A two-sided *P* < 0.05 was considered to indicate statistical significance. Data were analyzed using GraphPad Prism, v9.1.2 (GraphPad Software) and R software, v4.21 (R Foundation for Statistical Computing).

#### Missing data

There were 3.5–5% missing data in the cross-sectional cohort and 2–3% in the longitudinal cohort. The amount of missing data was distributed without focality with respect to individual data entries or time periods. In cases where data were missing, these entries were left blank and no forms of imputation were performed.

### Reporting summary

Further information on research design is available in the Nature Portfolio Reporting Summary linked to this article.

## Online content

Any methods, additional references, Nature Portfolio reporting summaries, source data, extended data, supplementary information, acknowledgements, peer review information; details of author contributions and competing interests; and statements of data and code availability are available at 10.1038/s41591-025-04134-3.

## Supplementary information

Supplementary Information Supplementary Tables 1 and 2 and Notes 1–3.

Reporting Summary

Supplementary Data 1 Sample data for the machine learning code.

## Source data

Source Data Fig. 1 Uncropped western blot scan for Extended Data Fig. 3a.

## Figures and Tables

**Extended Data Table 1:**
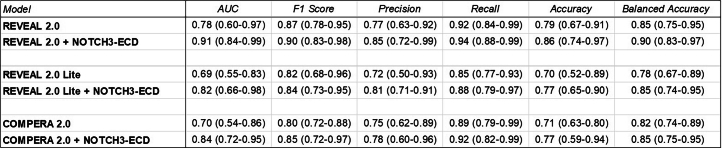
Machine learning metrics

The addition of serum NOTCH3-ECD to prognostic calculators, REVEAL 2.0, REVEAL 2.0 Lite and COMPERA 2.0, with machine learning (XGBoost)-improved 3-year mortality risk prediction in IPAH individuals. AUC = area under the curve; NOTCH3-ECD = NOTCH3 extracellular domain.

**Extended Data Table 2:**
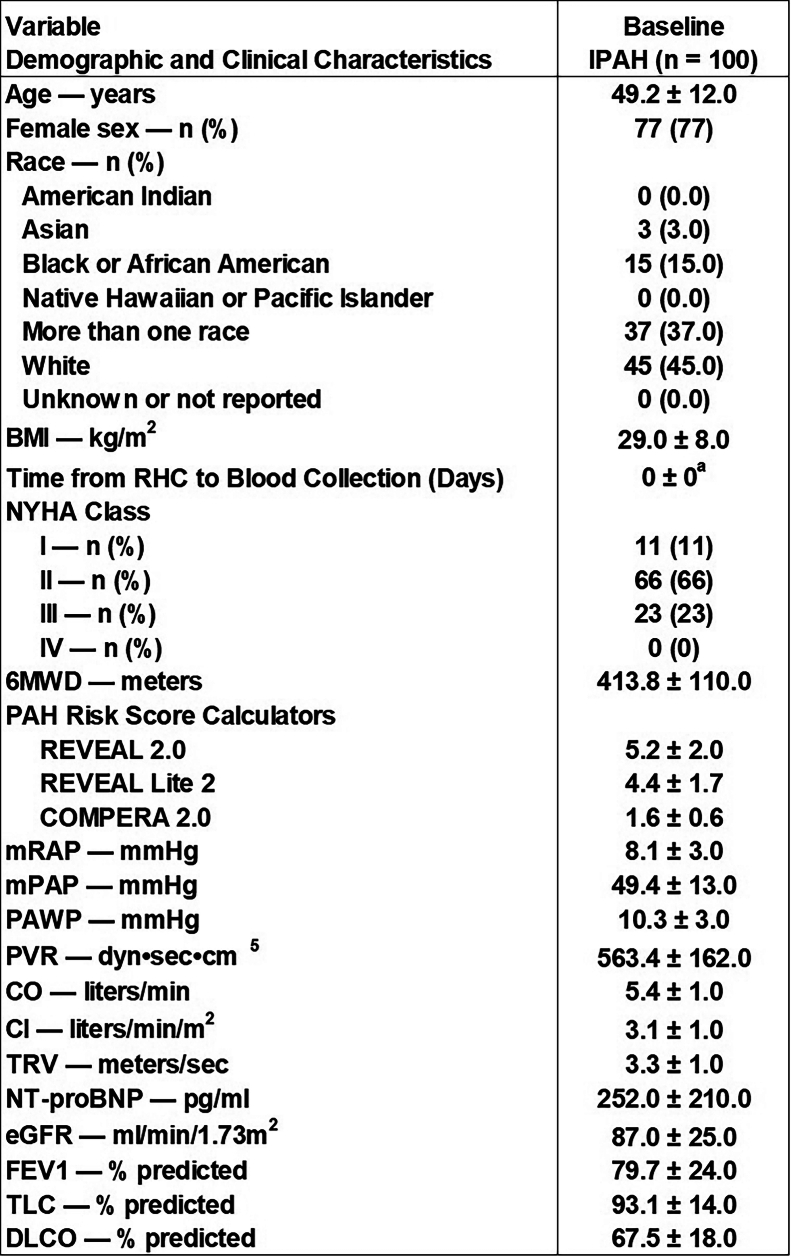
Treatment-naive IPAH longitudinal cohort

The values are expressed as mean ± s.d.

^a^Blood samples were taken at the time of right heart catheterization (RHC). IPAH = World Health Organization Group 1.1: idiopathic pulmonary arterial hypertension; n = number; BMI = body mass index; NYHA = New York Heart Association; 6MWD = 6-minute walk distance; mRAP = mean right atrial pressure; mPAP = mean pulmonary arterial pressure; PAWP = pulmonary arterial wedge pressure; PVR = pulmonary vascular resistance; CO = cardiac output; CI = cardiac index; TRV = tricuspid regurgitant velocity; NT-proBNP = n-terminal prohormone brain natriuretic peptide; eGFR = estimated glomerular filtration rate; FEV1 = forced expiratory volume in one second; TLC = total lung capacity; DLCO = diffusing capacity of the lungs for carbon monoxide.

## Data Availability

Restrictions apply to the availability of the in-house and external patient data (datasets from multiple institutions including UCSD, University of Arizona, Massachusetts General Hospital, Americas Hospital Guadalajara Mexico, University of Alabama, University of New Mexico, UCLA, Stanford, University of Cambridge and Mount Sinai), which were used with institutional permission through IRB approval and are thus not publicly available. Data that generated the results reported in this article will be made available to individual researchers by request to the corresponding author (P.A.T.: pthistlethwaite@ucsd.edu). Data may be requested for up to 10 years after publication. Requests will be evaluated based on institutional and departmental policies to determine whether the data requested are subject to intellectual property or patient privacy obligations. If approved, and after a formal data use agreement has been signed, data will be provided by the corresponding author through a secure web platform within 2 months of the request. Source data are provided with this paper.

## Code availability

All code used for the machine learning analysis is provided in Supplementary Note 1. The sample data are available as Supplementary Data 1. The ReadMe file and sample script for the machine learning model are also available in Supplementary Notes 2 and 3, respectively.
