# Supplementary material for: The NOTCH3 extracellular domain is a serum biomarker for pulmonary arterial hypertension
Source: Nat Med. 2026 Jan 9;32(1):306–17. doi: 10.1038/s41591-025-04134-3 (PMC12823441; doi:10.1038/s41591-025-04134-3)
Supplement: Supplementary file 1 — Supplementary Tables 1 and 2 and Notes 1–3. [file 41591_2025_4134_MOESM1_ESM.pdf]

nature medicine

Supplementary information https://doi.org/10.1038/s41591-025-04134-3

The NOTCH3 extracellular domain is a serum biomarker for pulmonary arterial hypertension

In the format provided by the authors and unedited

**Supplementary Information Table 1. NOTCH3-ECD Biomarker Study Eligibility Criteria for IPAH Individuals. Page 1.**
**Inclusion Criteria:**
Age ≥ 21 years<br>For individuals in the cross-sectional cohort, time from RHC or ECHO to blood collection to be within 30 days, with the following criteria:<br>Mean pulmonary arterial pressure > 20 mmHg<br>Pulmonary vascular resistance > 160 dynes·sec·cm^{-5} (2 wood units)<br>Pulmonary artery wedge pressure ≤ 15 mmHg<br>For individuals in the longitudinal cohort, blood collection to be at the time of RHC or ECHO, with the following criteria:<br>Mean pulmonary arterial pressure > 20 mmHg<br>Pulmonary vascular resistance > 160 dynes·sec·cm^{-5} (2 wood units)<br>Pulmonary artery wedge pressure ≤ 15 mmHg<br>Pulmonary function tests completed within one year prior to the first blood collection, with the following criteria:<br>Total lung capacity > 70%, or<br>FEV1/FVC ratio > 70% of the predicted value<br>High-resolution computed tomography showing no evidence of parenchymal lung disease, or masses in the lungs, mediastinum, or chest wall<br>Ventilation perfusion test with normal results or a negative CTPA<br>No investigator-identified contraindications for RHC throughout the study<br>Capability to follow the visit schedule and sign an informed consent
**Exclusion Criteria:**
Active or recent history of hepatitis viral infections<br>History of chronic liver diseases, classified as mild to end stage hepatic impairment based on MELD score<br>Liver transplant candidates or recipients<br>History of portal hypertension, congestive hepatopathy, or any of the following clinical laboratory values:<br>Serum AST and ALT levels exceeding 2 times the normal upper threshold<br>Serum total bilirubin level exceeding 1.5 times the normal upper threshold<br>Positive for HIV infection or absolute neutrophil count < 1500/mm^3<br>Individuals with first degree relatives diagnosed with heritable pulmonary arterial hypertension<br>History of pulmonary veno-occlusive disease (PVOD)<br>Individuals with history of scleroderma or a diagnosis of a connective tissue disorder<br>Individuals with a history of methamphetamine, fenfluramine use, or rapeseed oil ingestion<br>History of congenital heart defects<br>Individuals having received a lung or heart/lung transplant<br>History of constrictive pericarditis or a diagnosis of congestive or restrictive cardiomyopathy<br>Left ventricular ejection fraction < 45%<br>Known coronary lesion with > 50% stenosis in the LAD, RCA, LCx, diagonal branches, or obtuse marginal arteries, with or without symptoms<br>History of symptomatic coronary disease within 1 year prior to the first blood collection<br>History of atrial septostomy<br>Individuals with mitral, aortic, or tricuspid regurgitation (≥ 1+ regurgitation) at the time of first blood collection<br>History of aortic stenosis, mitral stenosis, or tricuspid stenosis

**Supplementary Information Table 1. NOTCH3-ECD Biomarker Study Eligibility Criteria for IPAH Individuals. Page 2.**
Acute decompensated heart failure or individuals on intravenous inotropes within 50 days prior to the first blood collection
Systolic blood pressure > 160 mmHg or diastolic blood pressure > 100 mmHg at rest
Systolic blood pressure < 80 mmHg at the time of first blood collection
History of chronic thromboembolic pulmonary hypertension with an abnormal ventilation-perfusion scan, CTPA, or prior pulmonary endarterectomy
History of non-PAH related autoimmune diseases, or positive autoantibody tests as outlined below:<br>Antinuclear antibody (ANA)<br>Anti-centromere antibody (ACA)<br>Antimitochondrial antibody (AMA)<br>Anti-double-stranded DNA antibody (Anti-dsDNA)<br>Anti topoisomerase I antibody (Anti-Scl-70)<br>Anti-Ro (SS-A) and Anti-La (SS-B) antibodies
History of opportunistic infections with a white blood cell count < 4000/mm3
History of active schistosomiasis infection at the time of first blood collection
History of systemic or local infections within 4 months prior to the first blood collection
Individuals with hemoglobin levels > 16 g/dl at the time of first blood collection
History of advanced parenchymal or interstitial lung diseases
History of severe obstructive or restrictive lung diseases
Diagnosis of severe obstructive sleep apnea or hypoventilation syndrome
History of developmental lung disorders
History of fibrosing mediastinitis
Individuals who have received new, adjusted, or stable doses of corticosteroids within 30 days prior to the time of first blood collection
Individuals who have resided at high altitude for > 3 months
Pregnant females
History of malignancy, excluding completely excised or treated basal cell carcinoma or cervical carcinoma in situ
History of clinically significant systemic diseases unrelated to PAH that could impede participation
Estimated GFR < 30 ml/min/1.73m^2
BMI > 40 kg/m^2
Enrollment in any clinical trial or study involving use of an investigational product within 3 months prior to the first blood collection
Non-compliance with study requirements
IPAH = World Health Organization Group 1.1: idiopathic pulmonary arterial hypertension, RHC = right heart catheterization, ECHO = echocardiogram, FEV1/FVC ratio = forced expiratory volume in 1 second/forced vital capacity ratio, CTPA = computed tomography pulmonary angiography, MELD = model for end stage liver disease, AST = aspartate aminotransferase, ALT = alanine aminotransferase, HIV = human immunodeficiency virus, LAD = left anterior descending, RCA = right coronary artery, LCx = left circumflex, GFR = glomerular filtration rate, BMI = body mass index.

Supplementary Information Table 2. Backwards Stepwise Regression

Step 1

Variable	*Coefficients*	*Standard Error*	*Beta*	*t*	*P-value > t*
**Age**	0.00393	0.00179	0.12477	2.19390	0.02895
**Sex**	-0.14299	0.05903	-0.12873	-2.42245	0.01597
**NOTCH3-ECD**	0.01190	0.00471	0.16619	2.52456	0.01206
**mRAP**	-0.00279	0.01004	-0.01804	-0.27822	0.78102
**6MWD**	0.00027	0.00020	0.07754	1.34319	0.18015
**PVR**	0.00016	0.00012	0.09337	1.37243	0.17088
**mPAP**	0.00085	0.00188	0.02966	0.45023	0.65285
**NT-proBNP**	0.00005	0.00002	0.13523	2.40353	0.01680
**NYHA Class**	0.07697	0.04083	0.11630	1.88498	0.06033
**eGFR**	0.00060	0.00096	0.03403	0.62579	0.53189
**PAWP**	0.00248	0.00637	0.02152	0.38903	0.69751
**SBP**	-0.00174	0.00147	-0.06577	-1.18250	0.23788
**HR**	0.00026	0.00194	0.00756	0.13537	0.89241
**CO**	-0.00219	0.01620	-0.00797	-0.13513	0.89259
**CI**	-0.00920	0.02795	-0.01949	-0.32901	0.74236
**Hospitalizations ≤ 6 mo**	-0.00094	0.04974	-0.00102	-0.01885	0.98498
**Pericardial Effusion**	-0.01935	0.05302	-0.01966	-0.36496	0.71538

Step 11

Variable	*Coefficients*	*Standard Error*	*Beta*	*t*	*P-value > t*
**Age**	0.00339	0.00163	0.10760	2.07738	0.03853
**Sex**	-0.14220	0.05755	-0.12802	-2.47078	0.01398
**NOTCH3-ECD**	0.01261	0.00433	0.17610	2.91205	0.00383
**6MWD**	0.00027	0.00019	0.07844	1.42494	0.15511
**PVR**	0.00019	0.00010	0.10916	1.90257	0.05796
**NT-proBNP**	0.00005	0.00002	0.13193	2.50133	0.01285
**NYHA Class**	0.07450	0.03866	0.11257	1.92725	0.05480

Backwards stepwise logistic regression to identify variables significantly associated with three-year mortality risk in IPAH individuals. NOTCH3-ECD = NOTCH3 extracellular domain, mRAP = mean right atrial pressure, 6MWD = 6-minute walk distance, PVR = pulmonary vascular resistance, mPAP = mean pulmonary artery pressure, NT-proBNP = n-terminal prohormone brain natriuretic peptide, NYHA = New York Heart Association, eGFR = estimated glomerular filtration rate, PAWP = pulmonary arterial wedge pressure, SBP = systolic blood pressure, HR = heart rate, CO = cardiac output, CI = cardiac index, mo = months.

```
## Supplementary Note 1. Machine Learning Model for IPAH Survival.

# Clear the workspace
rm(list = ls())

# Load necessary libraries ----------------------------------------------------
-----
# Data manipulation and preparation
library(dplyr)          # Data manipulation verbs (select, filter, etc.)
library(fastDummies)    # For creating dummy variables
library(readr)          # For reading CSV files efficiently

# Machine Learning Core
library(xgboost)        # Extreme Gradient Boosting algorithm

# Model Tuning
library(ParBayesianOptimization) # For hyperparameter tuning using
Bayesian Optimization
library(doParallel)     # For parallel processing during tuning

# Model Evaluation
library(caTools)        # For sample.split function (train/test
splitting)
library(ROCR)           # For creating prediction and performance objects
(AUC)
library(pROC)           # For ROC curve analysis and confidence intervals
library(MLmetrics)      # For various classification metrics (F1,
Precision, Recall, Accuracy)
library(caret)          # For confusionMatrix function (confusion matrix
and metrics)

# Plotting
library(ggplot2)        # For creating plots

# --- Configuration -----------------------------------------------------------
-----
# Set seed for reproducibility of random processes like splitting and
XGBoost internal randomness
set.seed(1502)

# --- Data Loading and Preparation ----------------------------------------
-----

# Load the dataset
# The CSV is expected to have a 'Mortality' column (dependent variable)
and other predictor columns (numeric and character/factor).
IPAHData <- read_csv("XXX")

# --- Data Preprocessing ---

# Define the dependent variable name
dependent_variable <- "Mortality"

# Identify character/factor columns to be converted to dummy variables
```

```
# Exclude the dependent variable
cols_to_dummy <- IPAHData %>%
  select_if(function(col) is.character(col) || is.factor(col)) %>%
  select(-any_of(dependent_variable)) %>%
  names()

# Create dummy variables for categorical features
# remove_first_dummy=TRUE helps avoid multicollinearity
# remove_selected_columns=TRUE removes the original categorical columns
after creating dummies
if (length(cols_to_dummy) > 0) {
  dataset_processed <- dummy_cols(IPAHData,
                              select_columns = cols_to_dummy,
                              remove_first_dummy = TRUE,
                              remove_selected_columns = TRUE)
} else {
  dataset_processed <- IPAHData
}

# Ensure the dependent variable is a factor; convert to 0/1 numeric
instead of yes/no
dataset_processed[[dependent_variable]] <-
as.factor(dataset_processed[[dependent_variable]])

# Reorder columns to place the dependent variable first (optional)
dv_col_index <- which(names(dataset_processed) == dependent_variable)
other_col_indices <- setdiff(1:ncol(dataset_processed), dv_col_index)
finaldataset <- dataset_processed[, c(dv_col_index, other_col_indices)]

# --- Train/Test Split ------------------------------------------------------
-----

# Split the dataset into training (80%) and testing (20%) sets
# Stratified split based on the 'Mortality' outcome to maintain
prevalence in both sets
split <- sample.split(finaldataset[[dependent_variable]], SplitRatio =
0.8)

training_set <- subset(finaldataset, split == TRUE)
test_set <- subset(finaldataset, split == FALSE)

print(paste("Training set size:", nrow(training_set)))
print(paste("Test set size:", nrow(test_set)))
print("Training set outcome distribution:")
print(table(training_set[[dependent_variable]]))
print("Test set outcome distribution:")
print(table(test_set[[dependent_variable]]))

# --- Prepare Data for XGBoost ------------------------------------------
-----

train.y <- as.numeric(training_set[[dependent_variable]]) - 1
test.y <- as.numeric(test_set[[dependent_variable]]) - 1
```

```
# Isolate the predictor variables (X) and convert them to a matrix
# Assumes excluding the dependent variable which is the FIRST column
after previous rearrangement
train.x <- as.matrix(training_set[, -1])
test.x <- as.matrix(test_set[, -1])

# --- Initial XGBoost Model (Finding Optimal Rounds) --------------------
-----

# Define initial XGBoost parameters (these will be tuned later)
# eta: learning rate
# max_depth: maximum tree depth
# subsample: fraction of observations sampled for each tree
# colsample_bytree: fraction of columns sampled for each tree
# min_child_weight: minimum sum of instance weight needed in a child
# eval_metric: metric used for evaluation (AUC for binary classification
in this case)
# objective: learning task objective (binary:logistic for binary
classification)
# booster: type of booster (gbtree for tree-based models)

parameters_initial <- list(eta = 0.1,
                           max_depth = 6,
                           subsample = 1.0,
                           colsample_bytree = 1.0,
                           min_child_weight = 1,
                           gamma = 0,
                           lambda = 1,
                           alpha = 0,
                           missing = NA,
                           eval_metric = "auc",
                           objective = "binary:logistic",
                           booster = "gbtree")

# Determine the number of CPU cores available for parallel processing
(optional)
n_cores <- detectCores()
n_threads_xgb <- max(1, n_cores - 1)

# Perform cross-validation (CV) to find the optimal number of boosting
rounds (nrounds)
# Uses the initial parameters and evaluates performance on validation
folds
print("Running initial XGBoost CV to find optimal boosting rounds...")
xgb_cv_initial <- xgb.cv(data = train.x,
                         label = train.y,
                         params = parameters_initial,
                         nthread = n_threads_xgb,
                         nrounds = 1000,
                         nfold = 10,
                         prediction = FALSE,
                         showsd = TRUE,
                         stratified = TRUE,
                         print_every_n = 100,
```

```
                         early_stopping_rounds = 20,
                         maximize = TRUE)

# Find the optimal number of rounds based on the best CV test AUC
numrounds_optimal <- xgb_cv_initial$best_iteration
print(paste("Optimal number of rounds found via CV:", numrounds_optimal))
print(paste("Best Test AUC during CV:",
xgb_cv_initial$evaluation_log$test_auc_mean[numrounds_optimal]))

# --- Hyperparameter Optimization (Bayesian Optimization) ---------------
-----

# Define the scoring function for Bayesian Optimization
scoring_function <- function(eta, gamma, max_depth, min_child_weight,
subsample, colsample_bytree, lambda, alpha) {


  dtrain <- xgb.DMatrix(train.x, label = train.y, missing = NA)

  # Define parameters
  params_cv <- list(
    eta = eta,
    max_depth = as.integer(max_depth),
    min_child_weight = min_child_weight,
    subsample = subsample,
    colsample_bytree = colsample_bytree,
    gamma = gamma,
    lambda = lambda,
    alpha = alpha,

    booster = "gbtree",
    objective = "binary:logistic",
    eval_metric = "auc",
    verbosity = 0
  )

  # Run XGBoost CV
  xgbcv <- xgb.cv(
    params = params_cv,
    data = dtrain,
    nfold = 5,
    nrounds = 500,
    prediction = FALSE,
    showsd = TRUE,
    early_stopping_rounds = 15,
    maximize = TRUE,
    stratified = TRUE,
    nthread = 2
  )

  # Return the best score and corresponding # of rounds
  return(
    list(
      Score = max(xgbcv$evaluation_log$test_auc_mean),
```

```
      nrounds = xgbcv$best_iteration
    )
  )
}

# Define the hyperparameter bounds
bounds <- list(
  eta = c(0.01, 0.3),
  gamma = c(0, 5),
  max_depth = c(3L, 10L),
  min_child_weight = c(1.0, 10.0),
  subsample = c(0.5, 1.0),
  colsample_bytree = c(0.5, 1.0),
  lambda = c(0.0, 5.0),
  alpha = c(0.0, 5.0)
)

# Set up parallel processing for Bayesian Optimization (optional)
num_cores_opt <- max(1, floor(detectCores() / 2))
print(paste("Setting up parallel backend for Bayesian Optimization with",
num_cores_opt, "cores."))
cl <- makeCluster(num_cores_opt)
registerDoParallel(cl)

clusterExport(cl, c('train.x', 'train.y', 'xgb.DMatrix'))
clusterEvalQ(cl, expr = { library(xgboost) })

# Run Bayesian Optimization
print("Starting Bayesian Optimization for hyperparameter tuning...")
time_optimization <- system.time(
  opt_obj <- bayesOpt(
    FUN = scoring_function,
    bounds = bounds,
    initPoints = 10,
    iters.n = 30,
    acq = "ucb",
    kappa = 2.576,
    parallel = TRUE
  )
)
stopCluster(cl)
registerDoSEQ()

print("Bayesian Optimization finished.")
print(paste("Time taken for optimization:",
round(time_optimization["elapsed"], 2), "seconds"))

# Collect the optimized parameters
best_params_tuned <- getBestPars(opt_obj)
print("Best hyperparameters found:")
print(best_params_tuned)

# --- Final Model Training with Tuned Parameters ------------------------
-----
```

```
# Prepare the final parameter list with the optimal parameters
params_tuned_final <- list(
    eta = best_params_tuned["eta"],
    max_depth = as.integer(best_params_tuned["max_depth"]),
    min_child_weight = best_params_tuned["min_child_weight"],
    subsample = best_params_tuned["subsample"],
    colsample_bytree = best_params_tuned["colsample_bytree"],
    gamma = best_params_tuned["gamma"],
    lambda = best_params_tuned["lambda"],
    alpha = best_params_tuned["alpha"],

    objective = "binary:logistic",
    eval_metric = "auc",
    booster = "gbtree",
    missing = NA
    )


print("Running final CV with tuned parameters to confirm optimal
rounds...")
xgb_cv_final <- xgb.cv(data = train.x,
                       label = train.y,
                       params = params_tuned_final,
                       nthread = n_threads_xgb,
                       nrounds = 1500,
                       nfold = 10,
                       prediction = FALSE,
                       showsd = TRUE,
                       stratified = TRUE,
                       print_every_n = 100,
                       early_stopping_rounds = 30,
                       maximize = TRUE)
numrounds_final <- xgb_cv_final$best_iteration
print(paste("Optimal number of rounds for tuned parameters:",
numrounds_final))
print(paste("Best Test AUC during final CV:",
xgb_cv_final$evaluation_log$test_auc_mean[numrounds_final]))


# Train the final XGBoost model on the training set using the optimized
parameters and round #
print("Training final XGBoost model on the full training set...")
fit_tuned <- xgboost(params = params_tuned_final,
                     data = train.x,
                     label = train.y,
                     nrounds = numrounds_final,
                     nthread = n_threads_xgb,
                     verbose = 1)

print("Final model training complete.")

# --- Model Evaluation on the Test Set ---------------------------------
----
```

```
# Predict probabilities on the test set
pred.xgb.tuned <- predict(fit_tuned, test.x, type = "response")

# --- Calculate Performance Metrics ---

# 1. AUC (Area Under the ROC Curve)
ROCpred.xgb.tuned <- ROCR::prediction(pred.xgb.tuned, test.y)
auc.xgb.tuned <- ROCR::performance(ROCpred.xgb.tuned, measure = "auc")
auc_value <- auc.xgb.tuned@y.values[[1]]
print(paste("Test Set AUC:", round(auc_value, 4)))

# Calculate 95% Confidence Interval for AUC using pROC
roc_obj_test <- pROC::roc(response = test.y, predictor = pred.xgb.tuned,
quiet = TRUE)
auc_ci <- pROC::ci.auc(roc_obj_test)
print(paste("Test Set AUC 95% CI:", round(auc_ci[1], 4), "-",
round(auc_ci[3], 4)))

# 2. Calculating other model metrics
predicted_labels <- ifelse(pred.xgb.tuned > 0.5, 1, 0)
accuracy <- MLmetrics::Accuracy(y_pred = predicted_labels, y_true =
test.y)
precision <- MLmetrics::Precision(y_pred = predicted_labels, y_true =
test.y)
recall <- MLmetrics::Recall(y_pred = predicted_labels, y_true = test.y)
f1_score <- MLmetrics::F1_Score(y_pred = predicted_labels, y_true =
test.y)

# Calculate Specificity (True Negative Rate)
# Specificity = TN / (TN + FP)
conf_matrix <- table(Actual = test.y, Predicted = predicted_labels)
if (!all(c("0", "1") %in% colnames(conf_matrix))) {
  if (!("0" %in% colnames(conf_matrix))) conf_matrix <- cbind("0" = c(0,
0), conf_matrix)
  if (!("1" %in% colnames(conf_matrix))) conf_matrix <-
cbind(conf_matrix, "1" = c(0, 0))
  conf_matrix <- conf_matrix[, c("0", "1")]
}
if (!all(c("0", "1") %in% rownames(conf_matrix))) {
 if (!("0" %in% rownames(conf_matrix))) conf_matrix <- rbind("0" = c(0,
0), conf_matrix)
 if (!("1" %in% rownames(conf_matrix))) conf_matrix <- rbind(conf_matrix,
"1" = c(0, 0))
 conf_matrix <- conf_matrix[c("0", "1"), ]
}

tn <- conf_matrix["0", "0"]
fp <- conf_matrix["0", "1"]
specificity <- tn / (tn + fp)
if (is.nan(specificity)) specificity <- 0

balanced_accuracy <- (recall + specificity) / 2
```

```
print(paste("Test Set Accuracy:", round(accuracy, 4)))
print(paste("Test Set Precision:", round(precision, 4)))
print(paste("Test Set Recall (Sensitivity):", round(recall, 4)))
print(paste("Test Set Specificity:", round(specificity, 4)))
print(paste("Test Set F1 Score:", round(f1_score, 4)))
print(paste("Test Set Balanced Accuracy:", round(balanced_accuracy, 4)))
print("Confusion Matrix (Actual vs Predicted):")
print(conf_matrix)


# --- Plot ROC Curve for the Test Set ---
print("Generating ROC curve for the test set...")

# Create data frame for ggplot
roc_data_test <- data.frame(
  fpr = pROC::roc(test.y, pred.xgb.tuned, quiet = TRUE)$specificities,
  tpr = pROC::roc(test.y, pred.xgb.tuned, quiet = TRUE)$sensitivities
)
# Need to reverse specificities for FPR (1 - specificity) and sort
roc_data_test$fpr <- 1 - roc_data_test$fpr
roc_data_test <- roc_data_test[order(roc_data_test$fpr,
roc_data_test$tpr), ]

roc_plot_test <- ggplot(roc_data_test, aes(x = fpr, y = tpr)) +
  geom_line(color = "blue", size = 1) +
  geom_abline(intercept = 0, slope = 1, linetype = "dashed", color =
"grey") +
  labs(
    title = "ROC Curve - Tuned XGBoost Model (Test Set)",
    x = "False Positive Rate (1 - Specificity)",
    y = "True Positive Rate (Sensitivity)",
    subtitle = paste("AUC =", round(auc_value, 3))
  ) +
  theme_minimal() +
  coord_fixed(ratio = 1) # Ensure aspect ratio is square

# Print the plot to the viewer/device
print(roc_plot_test)


# --- Save Final Model --------------------------------------------------
-----

# Save the trained model object for future use
model_save_path <- "xxx"
saveRDS(fit_tuned, file = model_save_path)
print(paste("Final tuned model saved to:", model_save_path))


# --- K-Fold CV on the Training Set (Using Tuned Parameters) ---
print("--- Starting K-Fold Cross-Validation on Training Set ---")
print("Purpose: Estimate performance variability using the tuned
hyperparameters.")
```

```
# Create k folds from the TRAINING data
# Using caret::createFolds for stratified folds based on the training
outcome
k_folds <- 10
folds <- createFolds(factor(train.y), k = k_folds, list = TRUE,
returnTrain = TRUE)

# Initialize vectors/lists to store metrics from each fold to calc the
mean and sd
cv_metrics <- list(
  AUC = numeric(k_folds),
  Accuracy = numeric(k_folds),
  Precision = numeric(k_folds),
  Recall = numeric(k_folds),
  Specificity = numeric(k_folds),
  F1_Score = numeric(k_folds),
  Balanced_Accuracy = numeric(k_folds)
)

calculate_fold_metrics <- function(actual_labels, predicted_probs) {

  predicted_labels <- ifelse(predicted_probs > 0.5, 1, 0)

  # Calculate metrics
  auc <- tryCatch(pROC::roc(actual_labels, predicted_probs, quiet =
TRUE)$auc, error = function(e) NA)
  accuracy <- tryCatch(MLmetrics::Accuracy(y_pred = predicted_labels,
y_true = actual_labels), error = function(e) NA)
  precision <- tryCatch(MLmetrics::Precision(y_pred = predicted_labels,
y_true = actual_labels), error = function(e) NA)
  recall <- tryCatch(MLmetrics::Recall(y_pred = predicted_labels, y_true
= actual_labels), error = function(e) NA)
  conf_matrix <- table(Actual = factor(actual_labels, levels=c(0,1)),
Predicted = factor(predicted_labels, levels=c(0,1)))
  tn <- conf_matrix["0", "0"]
  fp <- conf_matrix["0", "1"]
  specificity <- ifelse((tn + fp) == 0, NA, tn / (tn + fp))

  f1 <- tryCatch(MLmetrics::F1_Score(y_pred = predicted_labels, y_true =
actual_labels), error = function(e) NA)
  bal_accuracy <- ifelse(is.na(recall) || is.na(specificity), NA, (recall
+ specificity) / 2)

  return(list(AUC = auc, Accuracy = accuracy, Precision = precision,
Recall = recall, Specificity = specificity, F1_Score = f1,
Balanced_Accuracy = bal_accuracy))
}


# Loop through each fold
for (i in 1:k_folds) {
  print(paste("Processing Fold", i, "of", k_folds))

  train_indices <- folds[[i]]
```

```
  validation_indices <- setdiff(1:length(train.y), train_indices)

  train_fold_x <- train.x[train_indices, ]
  train_fold_y <- train.y[train_indices]
  validation_fold_x <- train.x[validation_indices, ]
  validation_fold_y <- train.y[validation_indices]

  model_fold <- xgboost(params = params_tuned_final,
                        data = train_fold_x,
                        label = train_fold_y,
                        nrounds = numrounds_final,
                        nthread = n_threads_xgb,
                        verbose = 0)

  # Predict probabilities on this fold's validation data
  predictions_fold <- predict(model_fold, validation_fold_x, type =
"response")

  # Calculate metrics for the current fold
  metrics_fold <- calculate_fold_metrics(actual_labels =
validation_fold_y, predicted_probs = predictions_fold)

  # Store metric values
  cv_metrics$AUC[i] <- metrics_fold$AUC
  cv_metrics$Accuracy[i] <- metrics_fold$Accuracy
  cv_metrics$Precision[i] <- metrics_fold$Precision
  cv_metrics$Recall[i] <- metrics_fold$Recall
  cv_metrics$Specificity[i] <- metrics_fold$Specificity
  cv_metrics$F1_Score[i] <- metrics_fold$F1_Score
  cv_metrics$Balanced_Accuracy[i] <- metrics_fold$Balanced_Accuracy

}

# Calculate mean and standard deviation across ALL folds
mean_cv_auc <- mean(cv_metrics$AUC, na.rm = TRUE)
sd_cv_auc <- sd(cv_metrics$AUC, na.rm = TRUE)
mean_cv_accuracy <- mean(cv_metrics$Accuracy, na.rm = TRUE)
sd_cv_accuracy <- sd(cv_metrics$Accuracy, na.rm = TRUE)
mean_cv_precision <- mean(cv_metrics$Precision, na.rm = TRUE)
sd_cv_precision <- sd(cv_metrics$Precision, na.rm = TRUE)
mean_cv_recall <- mean(cv_metrics$Recall, na.rm = TRUE)
sd_cv_recall <- sd(cv_metrics$Recall, na.rm = TRUE)
mean_cv_specificity <- mean(cv_metrics$Specificity, na.rm = TRUE)
sd_cv_specificity <- sd(cv_metrics$Specificity, na.rm = TRUE)
mean_cv_f1 <- mean(cv_metrics$F1_Score, na.rm = TRUE)
sd_cv_f1 <- sd(cv_metrics$F1_Score, na.rm = TRUE)
mean_cv_bal_accuracy <- mean(cv_metrics$Balanced_Accuracy, na.rm = TRUE)
sd_cv_bal_accuracy <- sd(cv_metrics$Balanced_Accuracy, na.rm = TRUE)

# Print summary of CV results
print("--- Cross-Validation Results Summary (on Training Set) ---")
print(paste("Mean CV AUC:", round(mean_cv_auc, 4), "+/-",
round(sd_cv_auc, 4)))
```

```
print(paste("Mean CV Accuracy:", round(mean_cv_accuracy, 4), "+/-",
round(sd_cv_accuracy, 4)))
print(paste("Mean CV Precision:", round(mean_cv_precision, 4), "+/-",
round(sd_cv_precision, 4)))
print(paste("Mean CV Recall (Sensitivity):", round(mean_cv_recall, 4),
"+/-", round(sd_cv_recall, 4)))
print(paste("Mean CV Specificity:", round(mean_cv_specificity, 4), "+/-",
round(sd_cv_specificity, 4)))
print(paste("Mean CV F1 Score:", round(mean_cv_f1, 4), "+/-",
round(sd_cv_f1, 4)))
print(paste("Mean CV Balanced Accuracy:", round(mean_cv_bal_accuracy, 4),
"+/-", round(sd_cv_bal_accuracy, 4)))
print("-----------------------------------------------------------")

# --- End of Script -----------------------------------------------------
-----
```

Supplementary Note 2 . ReadMe file

Machine Learning Model for IPAH Survival Prediction

1. Project Overview

This project contains an R script (ML_model_IPAH_survival.r) that builds, optimizes, and evaluates an **XGBoost machine learning model** to predict all cause mortality in patients with WHO Group 1.1 Idiopathic Pulmonary Arterial Hypertension (IPAH). The script performs the following key steps:

- Loads and preprocesses clinical data.
- Splits data into training and testing sets to predict overall survival.
- Uses Bayesian optimization to find the best hyperparameters for the XGBoost model.
- Trains a final model on the full training set using these optimal parameters.
- Evaluates the model's performance on the hold-out test set using metrics: AUC, F1 score, Accuracy, Precision, and Recall.
- Performs k-fold cross-validation to assess model stability.
- Generates a ROC curve plot for the test set.

2. System Requirements • R:
Version 4.0.0 or newer.

- **Operating System**: Windows, macOS, or Linux.

- **Hardware**: A multi-core CPU is helpful to accelerate the hyperparameter tuning step but is not strictly required.

- **R Packages**: The script requires the following R packages: "dplyr", "fastDummies", "readr", "xgboost", "ParBayesianOptimization", "doParallel", "caTools", "ROCR", "pROC", "MLmetrics", "caret", "ggplot2"

3. Installation Guide

1. Install R: Download and install R.

2. Install Packages: Open R or RStudio and run the following command in the console to install all required packages. install.packages(c("dplyr", "fastDummies", "readr", "xgboost", "ParBayesianOptimization", "doParallel", "caTools", "ROCR", "pROC", "MLmetrics", "caret", "ggplot2"))

4. Download Project Files: Download the ML_model_IPAH_survival.R script and the sample_data.csv file into the same project directory.

5. Demo: How to Run the Script

1. **Set Working Directory**: Open the ML_model_IPAH_survival.R script in RStudio. Set the working directory to the location of your files by going to Session > Set Working Directory > To Source File Location.

2. **Modify Data Loading**: In the script, find the line that reads the data: IPAHData <- read_csv("XXX") Change "XXX" to the name of the sample data file. IPAHData <- read_csv("sample_data.csv")

3. **Run the Script**: Execute the entire script.

4.1 Sample Dataset (sample_data.csv)

A small, simulated dataset is provided to demonstrate the script's functionality. It contains a Mortality column and several predictor variables.

4.2 Expected Output

After running the script, you will see output in the R console and a plot will be generated.

Console Output: The console will display progress and final evaluation metrics. The output will look similar to this (exact values will vary due to the randomness in data splitting and modeling):

```
[1] "Training set size: 75”
[1] "Test set size: 19”
...
[1] "Starting Bayesian Optimization for hyperparameter tuning..."
...
[1] "Best hyperparameters found:"
$eta
[1] 0.15
$max_depth
[1] 4
...
[1] "Training final XGBoost model on the full training set..."
...
```

1] "Test Set AUC: 0.6987"
[1] "Test Set AUC 95% CI: 0.4353 - 0.9621"
[1] "Test Set Accuracy: 0.6316"
[1] "Test Set Precision: 0.6875"
[1] "Test Set Recall (Sensitivity): 0.8462"
[1] "Test Set Specificity: 0.8462"
[1] "Test Set F1 Score: 0.7586"
[1] "Test Set Balanced Accuracy: 0.8462"
    Predicted
Actual 0 1
   0 11 2
1 5 1 ...

[1] "--- Cross-Validation Results Summary ---"
[1] "Mean CV AUC: 0.8817 +/- 0.1467"
[1] "Mean CV Accuracy: 0.825 +/- 0.0693" [1]
"Mean CV Precision: 0.831 +/- 0.1044"
[1] "Mean CV Recall (Sensitivity): 0.96 +/- 0.0843"
[1] "Mean CV Specificity: 0.96 +/- 0.0843"
[1] "Mean CV F1 Score: 0.8823 +/- 0.0348"
[1] "Mean CV Balanced Accuracy: 0.96 +/- 0.0843"
...```

Generated Plot: An ROC curve will be generated and displayed that illustrates the model's performance on the test set.

5. How to Use With Your Own Data

1. **Prepare your data**: Ensure your dataset is in a CSV file format in the same order and type case as the sample file. It must contain a column named `Mortality` with values like "Yes" and "No" (or can be adapted for 1/0).
2. **Update Script:** Change the filename in the `read_csv()`function to point to your new data file.
3. **Run:** Execute the script as described in the demo section. The model will train on your data, and the results and saved model (`final_model.rds`) will correspond to your dataset.

6. License

This project is licensed under the **MIT License.**

Supplementary Note 3. SAMPLE SCRIPT IPAH

```
# Clear the workspace
rm(list = ls())

# Load necessary libraries ---------------------------------------------------
-----
# Data manipulation and preparation
library(dplyr)          # Data manipulation verbs (select, filter, etc.)
library(fastDummies)    # For creating dummy variables
library(readr)          # For reading CSV files efficiently

# Machine Learning Core
library(xgboost)        # Extreme Gradient Boosting algorithm

# Model Tuning
library(ParBayesianOptimization) # For hyperparameter tuning using
Bayesian Optimization
library(doParallel)     # For parallel processing during tuning

# Model Evaluation
library(caTools)        # For sample.split function (train/test
splitting)
library(ROCR)           # For creating prediction and performance objects
(AUC)
library(pROC)           # For ROC curve analysis and confidence intervals
library(MLmetrics)      # For various classification metrics (F1,
Precision, Recall, Accuracy)
library(caret)          # For confusionMatrix function (confusion matrix
and metrics)

# Plotting
library(ggplot2)        # For creating plots

# --- Configuration ----------------------------------------------------------
-----
# Set seed for reproducibility of random processes like splitting and
XGBoost internal randomness
set.seed(1502)

# --- Data Loading and Preparation -------------------------------------------
-----

# Load the dataset
# The CSV is expected to have a 'Mortality' column (dependent variable)
and other predictor columns (numeric and character/factor).
IPAHData <- read_csv("sample_data.csv")

# --- Data Preprocessing ---

# Define the dependent variable name
dependent_variable <- "Mortality"

# Identify character/factor columns to be converted to dummy variables
```

```
# Exclude the dependent variable
cols_to_dummy <- IPAHData %>%
  select_if(function(col) is.character(col) || is.factor(col)) %>%
  select(-any_of(dependent_variable)) %>%
  names()

# Create dummy variables for categorical features
# remove_first_dummy=TRUE helps avoid multicollinearity
# remove_selected_columns=TRUE removes the original categorical columns
after creating dummies
if (length(cols_to_dummy) > 0) {
  dataset_processed <- dummy_cols(IPAHData,
                                  select_columns = cols_to_dummy,
                                  remove_first_dummy = TRUE,
                                  remove_selected_columns = TRUE)
} else {
  dataset_processed <- IPAHData
}

# Ensure the dependent variable is a factor; convert to 0/1 numeric
instead of yes/no
dataset_processed[[dependent_variable]] <-
as.factor(dataset_processed[[dependent_variable]])

# Reorder columns to place the dependent variable first (optional)
dv_col_index <- which(names(dataset_processed) == dependent_variable)
other_col_indices <- setdiff(1:ncol(dataset_processed), dv_col_index)
finaldataset <- dataset_processed[, c(dv_col_index, other_col_indices)]

# --- Train/Test Split ------------------------------------------------
-----

# Split the dataset into training (80%) and testing (20%) sets
# Stratified split based on the 'Mortality' outcome to maintain
prevalence in both sets
split <- sample.split(finaldataset[[dependent_variable]], SplitRatio =
0.8)

training_set <- subset(finaldataset, split == TRUE)
test_set <- subset(finaldataset, split == FALSE)

print(paste("Training set size:", nrow(training_set)))
print(paste("Test set size:", nrow(test_set)))
print("Training set outcome distribution:")
print(table(training_set[[dependent_variable]]))
print("Test set outcome distribution:")
print(table(test_set[[dependent_variable]]))

# --- Prepare Data for XGBoost ----------------------------------------
-----

train.y <- as.numeric(training_set[[dependent_variable]]) - 1
test.y <- as.numeric(test_set[[dependent_variable]]) - 1
```

```
# Isolate the predictor variables (X) and convert them to a matrix
# Assumes excluding the dependent variable which is the FIRST column
after previous rearrangement
train.x <- as.matrix(training_set[, -1])
test.x <- as.matrix(test_set[, -1])

# --- Initial XGBoost Model (Finding Optimal Rounds) -------------------
-----

# Define initial XGBoost parameters (these will be tuned later)
# eta: learning rate
# max_depth: maximum tree depth
# subsample: fraction of observations sampled for each tree
# colsample_bytree: fraction of columns sampled for each tree
# min_child_weight: minimum sum of instance weight needed in a child
# eval_metric: metric used for evaluation (AUC for binary classification
in this case)
# objective: learning task objective (binary:logistic for binary
classification)
# booster: type of booster (gbtree for tree-based models)

parameters_initial <- list(eta = 0.1,
                           max_depth = 6,
                           subsample = 1.0,
                           colsample_bytree = 1.0,
                           min_child_weight = 1,
                           gamma = 0,
                           lambda = 1,
                           alpha = 0,
                           missing = NA,
                           eval_metric = "auc",
                           objective = "binary:logistic",
                           booster = "gbtree")

# Determine the number of CPU cores available for parallel processing
(optional)
n_cores <- detectCores()
n_threads_xgb <- max(1, n_cores - 1)

# Perform cross-validation (CV) to find the optimal number of boosting
rounds (nrounds)
# Uses the initial parameters and evaluates performance on validation
folds
print("Running initial XGBoost CV to find optimal boosting rounds...")
xgb_cv_initial <- xgb.cv(data = train.x,
                         label = train.y,
                         params = parameters_initial,
                         nthread = n_threads_xgb,
                         nrounds = 1000,
                         nfold = 10,
                         prediction = FALSE,
                         showsd = TRUE,
                         stratified = TRUE,
                         print_every_n = 100,
```

```
                         early_stopping_rounds = 20,
                         maximize = TRUE)

# Find the optimal number of rounds based on the best CV test AUC
numrounds_optimal <- xgb_cv_initial$best_iteration
print(paste("Optimal number of rounds found via CV:", numrounds_optimal))
print(paste("Best Test AUC during CV:",
xgb_cv_initial$evaluation_log$test_auc_mean[numrounds_optimal]))

# --- Hyperparameter Optimization (Bayesian Optimization) ---------------
-----

# Define the scoring function for Bayesian Optimization
scoring_function <- function(eta, gamma, max_depth, min_child_weight,
subsample, colsample_bytree, lambda, alpha) {


  dtrain <- xgb.DMatrix(train.x, label = train.y, missing = NA)

  # Define parameters
  params_cv <- list(
    eta = eta,
    max_depth = as.integer(max_depth),
    min_child_weight = min_child_weight,
    subsample = subsample,
    colsample_bytree = colsample_bytree,
    gamma = gamma,
    lambda = lambda,
    alpha = alpha,

    booster = "gbtree",
    objective = "binary:logistic",
    eval_metric = "auc",
    verbosity = 0
  )

  # Run XGBoost CV
  xgbcv <- xgb.cv(
    params = params_cv,
    data = dtrain,
    nfold = 5,
    nrounds = 500,
    prediction = FALSE,
    showsd = TRUE,
    early_stopping_rounds = 15,
    maximize = TRUE,
    stratified = TRUE,
    nthread = 2
  )

  # Return the best score and corresponding # of rounds
  return(
    list(
      Score = max(xgbcv$evaluation_log$test_auc_mean),
```

```
      nrounds = xgbcv$best_iteration
    )
  )
}

# Define the hyperparameter bounds
bounds <- list(
  eta = c(0.01, 0.3),
  gamma = c(0, 5),
  max_depth = c(3L, 10L),
  min_child_weight = c(1.0, 10.0),
  subsample = c(0.5, 1.0),
  colsample_bytree = c(0.5, 1.0),
  lambda = c(0.0, 5.0),
  alpha = c(0.0, 5.0)
)

# Set up parallel processing for Bayesian Optimization (optional)
num_cores_opt <- max(1, floor(detectCores() / 2))
print(paste("Setting up parallel backend for Bayesian Optimization with",
num_cores_opt, "cores."))
cl <- makeCluster(num_cores_opt)
registerDoParallel(cl)

clusterExport(cl, c('train.x', 'train.y', 'xgb.DMatrix'))
clusterEvalQ(cl, expr = { library(xgboost) })

# Run Bayesian Optimization
print("Starting Bayesian Optimization for hyperparameter tuning...")
time_optimization <- system.time(
  opt_obj <- bayesOpt(
    FUN = scoring_function,
    bounds = bounds,
    initPoints = 10,
    iters.n = 30,
    acq = "ucb",
    kappa = 2.576,
    parallel = TRUE
  )
)
stopCluster(cl)
registerDoSEQ()

print("Bayesian Optimization finished.")
print(paste("Time taken for optimization:",
round(time_optimization["elapsed"], 2), "seconds"))

# Collect the optimized parameters
best_params_tuned <- getBestPars(opt_obj)
print("Best hyperparameters found:")
print(best_params_tuned)

# --- Final Model Training with Tuned Parameters -----------------------
-----
```

```
# Prepare the final parameter list with the optimal parameters
params_tuned_final <- list(
  eta = best_params_tuned["eta"],
  max_depth = as.integer(best_params_tuned["max_depth"]),
  min_child_weight = best_params_tuned["min_child_weight"],
  subsample = best_params_tuned["subsample"],
  colsample_bytree = best_params_tuned["colsample_bytree"],
  gamma = best_params_tuned["gamma"],
  lambda = best_params_tuned["lambda"],
  alpha = best_params_tuned["alpha"],

  objective = "binary:logistic",
  eval_metric = "auc",
  booster = "gbtree",
  missing = NA
)


print("Running final CV with tuned parameters to confirm optimal
rounds...")
xgb_cv_final <- xgb.cv(data = train.x,
                       label = train.y,
                       params = params_tuned_final,
                       nthread = n_threads_xgb,
                       nrounds = 1500,
                       nfold = 10,
                       prediction = FALSE,
                       showsd = TRUE,
                       stratified = TRUE,
                       print_every_n = 100,
                       early_stopping_rounds = 30,
                       maximize = TRUE)
numrounds_final <- xgb_cv_final$best_iteration
print(paste("Optimal number of rounds for tuned parameters:",
numrounds_final))
print(paste("Best Test AUC during final CV:",
xgb_cv_final$evaluation_log$test_auc_mean[numrounds_final]))


# Train the final XGBoost model on the training set using the optimized
parameters and round #
print("Training final XGBoost model on the full training set...")
fit_tuned <- xgboost(params = params_tuned_final,
                     data = train.x,
                     label = train.y,
                     nrounds = numrounds_final,
                     nthread = n_threads_xgb,
                     verbose = 1)

print("Final model training complete.")

# --- Model Evaluation on the Test Set ---------------------------------
----
```

```
# Predict probabilities on the test set
pred.xgb.tuned <- predict(fit_tuned, test.x, type = "response")

# --- Calculate Performance Metrics ---

# 1. AUC (Area Under the ROC Curve)
ROCpred.xgb.tuned <- ROCR::prediction(pred.xgb.tuned, test.y)
auc.xgb.tuned <- ROCR::performance(ROCpred.xgb.tuned, measure = "auc")
auc_value <- auc.xgb.tuned@y.values[[1]]
print(paste("Test Set AUC:", round(auc_value, 4)))

# Calculate 95% Confidence Interval for AUC using pROC
roc_obj_test <- pROC::roc(response = test.y, predictor = pred.xgb.tuned,
quiet = TRUE)
auc_ci <- pROC::ci.auc(roc_obj_test)
print(paste("Test Set AUC 95% CI:", round(auc_ci[1], 4), "-",
round(auc_ci[3], 4)))

# 2. Calculating other model metrics
predicted_labels <- ifelse(pred.xgb.tuned > 0.5, 1, 0)
accuracy <- MLmetrics::Accuracy(y_pred = predicted_labels, y_true =
test.y)
precision <- MLmetrics::Precision(y_pred = predicted_labels, y_true =
test.y)
recall <- MLmetrics::Recall(y_pred = predicted_labels, y_true = test.y)
f1_score <- MLmetrics::F1_Score(y_pred = predicted_labels, y_true =
test.y)

# Calculate Specificity (True Negative Rate)
# Specificity = TN / (TN + FP)
conf_matrix <- table(Actual = test.y, Predicted = predicted_labels)
if (!all(c("0", "1") %in% colnames(conf_matrix))) {
  if (!("0" %in% colnames(conf_matrix))) conf_matrix <- cbind("0" = c(0,
0), conf_matrix)
  if (!("1" %in% colnames(conf_matrix))) conf_matrix <-
cbind(conf_matrix, "1" = c(0, 0))
  conf_matrix <- conf_matrix[, c("0", "1")]
}
if (!all(c("0", "1") %in% rownames(conf_matrix))) {
  if (!("0" %in% rownames(conf_matrix))) conf_matrix <- rbind("0" = c(0,
0), conf_matrix)
  if (!("1" %in% rownames(conf_matrix))) conf_matrix <-
rbind(conf_matrix, "1" = c(0, 0))
  conf_matrix <- conf_matrix[c("0", "1"), ]
}

tn <- conf_matrix["0", "0"]
fp <- conf_matrix["0", "1"]
specificity <- tn / (tn + fp)
if (is.nan(specificity)) specificity <- 0

balanced_accuracy <- (recall + specificity) / 2
```

```r
print(paste("Test Set Accuracy:", round(accuracy, 4)))
print(paste("Test Set Precision:", round(precision, 4)))
print(paste("Test Set Recall (Sensitivity):", round(recall, 4)))
print(paste("Test Set Specificity:", round(specificity, 4)))
print(paste("Test Set F1 Score:", round(f1_score, 4)))
print(paste("Test Set Balanced Accuracy:", round(balanced_accuracy, 4)))
print("Confusion Matrix (Actual vs Predicted):")
print(conf_matrix)


# --- Plot ROC Curve for the Test Set ---
print("Generating ROC curve for the test set...")

# Create data frame for ggplot
roc_data_test <- data.frame(
  fpr = pROC::roc(test.y, pred.xgb.tuned, quiet = TRUE)$specificities,
  tpr = pROC::roc(test.y, pred.xgb.tuned, quiet = TRUE)$sensitivities
)
# Need to reverse specificities for FPR (1 - specificity) and sort
roc_data_test$fpr <- 1 - roc_data_test$fpr
roc_data_test <- roc_data_test[order(roc_data_test$fpr,
roc_data_test$tpr), ]

roc_plot_test <- ggplot(roc_data_test, aes(x = fpr, y = tpr)) +
  geom_line(color = "blue", size = 1) +
  geom_abline(intercept = 0, slope = 1, linetype = "dashed", color =
"grey") +
  labs(
    title = "ROC Curve - Tuned XGBoost Model (Test Set)",
    x = "False Positive Rate (1 - Specificity)",
    y = "True Positive Rate (Sensitivity)",
    subtitle = paste("AUC =", round(auc_value, 3))
  ) +
  theme_minimal() +
  coord_fixed(ratio = 1) # Ensure aspect ratio is square

# Print the plot to the viewer/device
print(roc_plot_test)


# --- Save Final Model --------------------------------------------------
-----

# Save the trained model object for future use
model_save_path <- "xxx"
saveRDS(fit_tuned, file = model_save_path)
print(paste("Final tuned model saved to:", model_save_path))


# --- K-Fold CV on the Training Set (Using Tuned Parameters) ---
print("--- Starting K-Fold Cross-Validation on Training Set ---")
print("Purpose: Estimate performance variability using the tuned
hyperparameters.")
```

```
# Create k folds from the TRAINING data
# Using caret::createFolds for stratified folds based on the training
outcome
k_folds <- 10
folds <- createFolds(factor(train.y), k = k_folds, list = TRUE,
returnTrain = TRUE)

# Initialize vectors/lists to store metrics from each fold to calc the
mean and sd
cv_metrics <- list(
  AUC = numeric(k_folds),
  Accuracy = numeric(k_folds),
  Precision = numeric(k_folds),
  Recall = numeric(k_folds),
  Specificity = numeric(k_folds),
  F1_Score = numeric(k_folds),
  Balanced_Accuracy = numeric(k_folds)
)

calculate_fold_metrics <- function(actual_labels, predicted_probs) {

  predicted_labels <- ifelse(predicted_probs > 0.5, 1, 0)

  # Calculate metrics
  auc <- tryCatch(pROC::roc(actual_labels, predicted_probs, quiet =
TRUE)$auc, error = function(e) NA)
  accuracy <- tryCatch(MLmetrics::Accuracy(y_pred = predicted_labels,
y_true = actual_labels), error = function(e) NA)
  precision <- tryCatch(MLmetrics::Precision(y_pred = predicted_labels,
y_true = actual_labels), error = function(e) NA)
  recall <- tryCatch(MLmetrics::Recall(y_pred = predicted_labels, y_true
= actual_labels), error = function(e) NA)
  conf_matrix <- table(Actual = factor(actual_labels, levels=c(0,1)),
Predicted = factor(predicted_labels, levels=c(0,1)))
  tn <- conf_matrix["0", "0"]
  fp <- conf_matrix["0", "1"]
  specificity <- ifelse((tn + fp) == 0, NA, tn / (tn + fp))

  f1 <- tryCatch(MLmetrics::F1_Score(y_pred = predicted_labels, y_true =
actual_labels), error = function(e) NA)
  bal_accuracy <- ifelse(is.na(recall) || is.na(specificity), NA, (recall
+ specificity) / 2)

  return(list(AUC = auc, Accuracy = accuracy, Precision = precision,
Recall = recall, Specificity = specificity, F1_Score = f1,
Balanced_Accuracy = bal_accuracy))
}


# Loop through each fold
for (i in 1:k_folds) {
  print(paste("Processing Fold", i, "of", k_folds))

  train_indices <- folds[[i]]
```

```
  validation_indices <- setdiff(1:length(train.y), train_indices)

  train_fold_x <- train.x[train_indices, ]
  train_fold_y <- train.y[train_indices]
  validation_fold_x <- train.x[validation_indices, ]
  validation_fold_y <- train.y[validation_indices]

  model_fold <- xgboost(params = params_tuned_final,
                        data = train_fold_x,
                        label = train_fold_y,
                        nrounds = numrounds_final,
                        nthread = n_threads_xgb,
                        verbose = 0)

  # Predict probabilities on this fold's validation data
  predictions_fold <- predict(model_fold, validation_fold_x, type =
"response")

  # Calculate metrics for the current fold
  metrics_fold <- calculate_fold_metrics(actual_labels =
validation_fold_y, predicted_probs = predictions_fold)

  # Store metric values
  cv_metrics$AUC[i] <- metrics_fold$AUC
  cv_metrics$Accuracy[i] <- metrics_fold$Accuracy
  cv_metrics$Precision[i] <- metrics_fold$Precision
  cv_metrics$Recall[i] <- metrics_fold$Recall
  cv_metrics$Specificity[i] <- metrics_fold$Specificity
  cv_metrics$F1_Score[i] <- metrics_fold$F1_Score
  cv_metrics$Balanced_Accuracy[i] <- metrics_fold$Balanced_Accuracy

}

# Calculate mean and standard deviation across ALL folds
mean_cv_auc <- mean(cv_metrics$AUC, na.rm = TRUE)
sd_cv_auc <- sd(cv_metrics$AUC, na.rm = TRUE)
mean_cv_accuracy <- mean(cv_metrics$Accuracy, na.rm = TRUE)
sd_cv_accuracy <- sd(cv_metrics$Accuracy, na.rm = TRUE)
mean_cv_precision <- mean(cv_metrics$Precision, na.rm = TRUE)
sd_cv_precision <- sd(cv_metrics$Precision, na.rm = TRUE)
mean_cv_recall <- mean(cv_metrics$Recall, na.rm = TRUE)
sd_cv_recall <- sd(cv_metrics$Recall, na.rm = TRUE)
mean_cv_specificity <- mean(cv_metrics$Specificity, na.rm = TRUE)
sd_cv_specificity <- sd(cv_metrics$Specificity, na.rm = TRUE)
mean_cv_f1 <- mean(cv_metrics$F1_Score, na.rm = TRUE)
sd_cv_f1 <- sd(cv_metrics$F1_Score, na.rm = TRUE)
mean_cv_bal_accuracy <- mean(cv_metrics$Balanced_Accuracy, na.rm = TRUE)
sd_cv_bal_accuracy <- sd(cv_metrics$Balanced_Accuracy, na.rm = TRUE)

# Print summary of CV results
print("--- Cross-Validation Results Summary (on Training Set) ---")
print(paste("Mean CV AUC:", round(mean_cv_auc, 4), "+/-",
round(sd_cv_auc, 4)))
```

```
print(paste("Mean CV Accuracy:", round(mean_cv_accuracy, 4), "+/-",
round(sd_cv_accuracy, 4)))
print(paste("Mean CV Precision:", round(mean_cv_precision, 4), "+/-",
round(sd_cv_precision, 4)))
print(paste("Mean CV Recall (Sensitivity):", round(mean_cv_recall, 4),
"+/-", round(sd_cv_recall, 4)))
print(paste("Mean CV Specificity:", round(mean_cv_specificity, 4), "+/-",
round(sd_cv_specificity, 4)))
print(paste("Mean CV F1 Score:", round(mean_cv_f1, 4), "+/-",
round(sd_cv_f1, 4)))
print(paste("Mean CV Balanced Accuracy:", round(mean_cv_bal_accuracy, 4),
"+/-", round(sd_cv_bal_accuracy, 4)))
print("-----------------------------------------------------------")

# --- End of Script -----------------------------------------------------
-----
```
