# Supplementary material for: The NOTCH3 extracellular domain is a serum biomarker for pulmonary arterial hypertension
Source: Nat Med. 2026 Jan 9;32(1):306–17. doi: 10.1038/s41591-025-04134-3 (PMC12823441; doi:10.1038/s41591-025-04134-3)
Supplement: Supplementary file 2 — Reporting Summary [file 41591_2025_4134_MOESM2_ESM.pdf]

nature portfolio

Corresponding author(s): Patricia A. Thistlethwaite

Last updated by author(s): 11/03/2025

Reporting Summary

Nature Portfolio wishes to improve the reproducibility of the work that we publish. This form provides structure for consistency and transparency in reporting. For further information on Nature Portfolio policies, see our Editorial Policies and the Editorial Policy Checklist.

Statistics

For all statistical analyses, confirm that the following items are present in the figure legend, table legend, main text, or Methods section.

n/a	Confirmed	
☐	☒	The exact sample size (*n*) for each experimental group/condition, given as a discrete number and unit of measurement
☐	☒	A statement on whether measurements were taken from distinct samples or whether the same sample was measured repeatedly
☐	☒	The statistical test(s) used AND whether they are one- or two-sided<br>*Only common tests should be described solely by name; describe more complex techniques in the Methods section.*
☐	☒	A description of all covariates tested
☐	☒	A description of any assumptions or corrections, such as tests of normality and adjustment for multiple comparisons
☐	☒	A full description of the statistical parameters including central tendency (e.g. means) or other basic estimates (e.g. regression coefficient) AND variation (e.g. standard deviation) or associated estimates of uncertainty (e.g. confidence intervals)
☐	☒	For null hypothesis testing, the test statistic (e.g. *F*, *t*, *r*) with confidence intervals, effect sizes, degrees of freedom and *P* value noted<br>*Give P values as exact values whenever suitable.*
☒	☐	For Bayesian analysis, information on the choice of priors and Markov chain Monte Carlo settings
☒	☐	For hierarchical and complex designs, identification of the appropriate level for tests and full reporting of outcomes
☒	☐	Estimates of effect sizes (e.g. Cohen's *d*, Pearson's *r*), indicating how they were calculated

Our web collection on statistics for biologists contains articles on many of the points above.

Software and code

Policy information about availability of computer code

Data collection	N/A
Data analysis	Data were analyzed with GraphPad Prism, version 9.1.2 (GraphPad Software, San Diego, CA) and R software, version 4.21 (R Foundation for Statistical Computing, Vienna, Austria). For machine learning analyses, Xgboost was used. Reference: Chen T, Guestrin C. Xgboost: A scalable tree boosting system. Proceedings of the 22nd ACM SIGKDD International Conference on Knowledge Discovery and Data Mining 2016 (p. 785-794).

For manuscripts utilizing custom algorithms or software that are central to the research but not yet described in published literature, software must be made available to editors and reviewers. We strongly encourage code deposition in a community repository (e.g. GitHub). See the Nature Portfolio guidelines for submitting code & software for further information.

Data

Policy information about availability of data

All manuscripts must include a data availability statement. This statement should provide the following information, where applicable:

- Accession codes, unique identifiers, or web links for publicly available datasets
- A description of any restrictions on data availability
- For clinical datasets or third party data, please ensure that the statement adheres to our policy

DATA AVAILABILITY

Restrictions apply to the availability of the in-house and external patient data (datasets from multiple institutions including UCSD, University of Arizona, Massachusetts General Hospital, Americas Hospital Guadalajara Mexico, University of Alabama, University of New Mexico, UCLA, Stanford, University of Cambridge, Mount Sinai), which were used with institutional permission through IRB approval, and are thus not publicly available.

Data that generated the results reported in this article will be made available to individual researchers by request to the corresponding author (P.A.T. pthistlethwaite@ucsd.edu). Data may be requested for up to 10 years after publication. Requests will be evaluated based on institutional and departmental policies to determine whether the data requested is subject to intellectual property or patient privacy obligations. If approved and after a formal data use agreement is signed, data will be provided by the corresponding author through a secure web platform within 2 months of request.

CODE AVAILABILITY
All code used for the machine learning analysis is provided in Supplementary Note 1 and the sample data are available in Supplementary Data . The ReadMe file and sample script for the machine learning model are also available in Supplementary Notes 2 and 3 respectively.

Research involving human participants, their data, or biological material

Policy information about studies with human participants or human data. See also policy information about sex, gender (identity/presentation), and sexual orientation and race, ethnicity and racism.

Reporting on sex and gender	Sex (biological attribute) was obtained from the electronic health record. The clinical data, including sex, is shown in Table 1 and Extended Data Table 2. Analysis of serum NOTCH3-ECD levels in relation to sex is included in Extended Data Figure 6.
Reporting on race, ethnicity, or other socially relevant groupings	Race was reported in Table 1 and Extended Data Table 2. Ethnicity was not available in the electronic medical health records of patients studied, thus it is not reported.
Population characteristics	Population characteristics are shown in Table 1 and Extended DataTable 2.
Recruitment	Consecutive IPAH and control patients from Boston, Phoenix, and San Diego were recruited to form the cross-sectional cohort. Participants were recruited by a PH physician at each site based on the study eligibility criteria. A second longitudinal cohort of newly diagnosed, treatment-naïve IPAH patients were recruited from UCSD and the University of Arizona.
Ethics oversight	All studies were approved by the UCSD Human Subjects Program and relevant IRB or ethics committees of all participating institutions.

Note that full information on the approval of the study protocol must also be provided in the manuscript.

Field-specific reporting

Please select the one below that is the best fit for your research. If you are not sure, read the appropriate sections before making your selection.

☒ Life sciences ☐ Behavioural & social sciences ☐ Ecological, evolutionary & environmental sciences

For a reference copy of the document with all sections, see nature.com/documents/nr-reporting-summary-flat.pdf

Life sciences study design

All studies must disclose on these points even when the disclosure is negative.

Sample size	This was an exploratory biomarker study of sera from IPAH patients. 341 IPAH and 376 control patients comprised the cross-sectional cohorts. 100 newly diagnosed, treatment-naïve IPAH patients comprised the longitudinal cohort. All the participants provided informed consent for this study.<br>A) A sample size of 341 individuals with IPAH and 376 individuals without PH was calculated to provide 90% power to detect a minimum effect size of 0.27 for the difference in serum NOTCH3-ECD levels between the two groups with a two-sided alpha = 0.05.<br>B) Sample numbers for secondary comparative analyses were determined by availability of serum samples over the 9 years of collection from multiple participating institutions, UCSD, and the UCSD Biorepository.<br>C) Sample numbers for longitudinal analysis were determined by availability of serum samples collected at three timepoints for each patient over a 6-year period.
Data exclusions	Only patients with WHO Group 1.1 IPAH and control patients were included in the primary analysis.
Replication	No comparable replication data sets were available.
Randomization	No randomization was performed.
Blinding	A) Serum samples from PAH and non-PH control groups in longitudinal and cross-sectional cohorts were blinded to the investigators. This is written in the Methods.

B) Serum samples for Western blot analysis were blinded to the investigators. This is written in the Methods.
C) Serum samples from individuals with other types of WHO Group PH and other types of WHO Group 1.1 PAH were not blinded, due to the small number of samples in each group and coordination of having samples sent from multiple institutions.
D) Serum samples from individuals with diseases know to express NOTCH3 and non-PH vasculitides were not blinded, as they were supplied by the UCSD Biorepository.

Reporting for specific materials, systems and methods

We require information from authors about some types of materials, experimental systems and methods used in many studies. Here, indicate whether each material, system or method listed is relevant to your study. If you are not sure if a list item applies to your research, read the appropriate section before selecting a response.

Materials & experimental systems

n/a	Involved in the study
☐	☒ Antibodies
☒	☐ Eukaryotic cell lines
☒	☐ Palaeontology and archaeology
☒	☐ Animals and other organisms
☒	☐ Clinical data
☒	☐ Dual use research of concern
☒	☐ Plants

Methods

n/a	Involved in the study
☒	☐ ChIP-seq
☒	☐ Flow cytometry
☒	☐ MRI-based neuroimaging

Antibodies

Antibodies used

The antibodies used for Western blotting were human anti-NOTCH3-ECD antibody (Clone 2G8; Catalog no. MABF937, Sigma-Aldrich, St. Louis, MO; dilution 1:1000), goat polyclonal anti-rat secondary antibody (Catalog no. 31470, Thermo Fisher Scientific, Waltham, MA; dilution 1:5000), rabbit polyclonal anti-transferrin antibody (Catalog no. PA527306, Thermo Fisher Scientific; dilution 1:1000), and horseradish peroxidase (HRP)-conjugated goat polyclonal anti-rabbit IgG antibody (Catalog no. 31460, Thermo Fisher Scientific dilution 1: 5000). For immunoprecipitation experiments, primary antibodies were used at a concentration of 6 μg of antibody per 1 mg of total protein.

Validation

For validation, please see the citations listed in the Sigma-Aldrich and Thermo Fisher Scientific catalog for each antibody.

Plants

Seed stocks

N/A

Novel plant genotypes

N/A

Authentication

N/A
