# Supplementary figures and images for: The NOTCH3 extracellular domain is a serum biomarker for pulmonary arterial hypertension
Source: Nat Med. 2026 Jan 9;32(1):306–17. doi: 10.1038/s41591-025-04134-3 (PMC12823441; doi:10.1038/s41591-025-04134-3)

# Source Data 1

Uncropped scans for Extended Data Fig 3a :

**NOTCH3-ECD**  
(235 kDa)

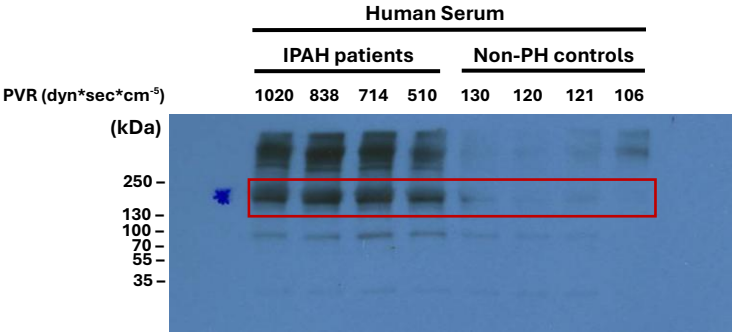

**TRANSFERRIN**  
75 (kDa)

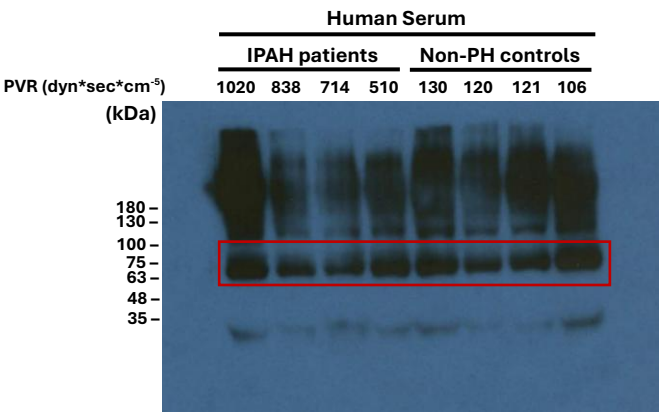

Supplement: Supplementary file 4 — Uncropped western blot scan for Extended Data Fig. 3a. [file 41591_2025_4134_MOESM4_ESM.pdf]
